# Non-surgical treatment of cyclosporin A-induced gingival overgrowth: A systematic review and meta-analysis

**DOI:** 10.1097/MD.0000000000043434

**Published:** 2025-07-18

**Authors:** Jiaan Hu, Yuting Ruan, Nanzhen Lin, Ying Zhang, Xinyi Mao, Junyi Bai, Xue Yang, Shan Liu, Xinxin Lv

**Affiliations:** a Department of Stomatology, The First Affiliated Hospital of Zhejiang Chinese Medical University (Zhejiang Provincial Hospital of Chinese Medicine), Hangzhou, China; b The First Clinical Medical College of Zhejiang University, Hangzhou, China; c School of Stomatology, Zhejiang Chinese Medical University, Hangzhou, China; d Department of Science and Development, The Second Affiliated Hospital of Zhejiang University School of Medicine, Hangzhou, China; e The First Clinical Medical College of Zhejiang Chinese Medical University, Hangzhou, China; f School of Stomatology, Dalian Medical University, Dalian, China; g Center of Clinical Evaluation, The First Affiliated Hospital of Zhejiang Chinese Medical University (Zhejiang Provincial Hospital of Chinese Medicine), Hangzhou, China.

**Keywords:** gingival overgrowth, meta-analysis, oral medicine, therapeutics

## Abstract

**Background:**

Cyclosporin A-induced (CsA-induced) gingival overgrowth (GO) is a common side effect affecting many patients. While surgical intervention is often considered the primary treatment, the effectiveness of nonsurgical periodontal therapy for CsA-induced GO remains unclear. This meta-analysis aimed to assess the efficacy of nonsurgical periodontal treatment in CsA-induced GO.

**Methods:**

Articles were retrieved from 7 databases (Cochrane Library, PubMed, EMBASE, China National Knowledge Infrastructure, Chinese Academic Database, Clinical Trials Database, and Grey Literature Database) until November 5th, 2024. Randomized controlled trials (RCTs) or pre–post studies containing patients with GO caused by cyclosporine who received nonsurgical periodontal treatment were included. The protocol was published in International Prospective Register of Systematic Reviews with the ID CRD42024570280.

**Results:**

A total of 13 studies were included. Five studies were RCTs comparing nonsurgical periodontal treatment groups and control groups among patients with GO; the other 8 studies were single group pre–post studies. Meta-analysis of RCTs demonstrated the hypertrophy index (standardized mean difference [SMD] = −1.14; 95% confidence intervals [CI]: [−1.59, −0.69]) and plaque index (SMD = −1.06, 95% CI: [−2.04, −0.07]) were significantly lower in the nonsurgical treatment groups than those in the control groups. Meta-analysis of single group pre–post studies showed that the levels of hypertrophy index (SMD = −1.49; 95% CI: [−2.92, −0.06]), probing depth (SMD = −1.52, 95% CI: [−2.46, −0.58]) and plaque index (SMD = −1.53, 95% CI: [−2.18, −0.88]) decreased significantly after treatment. Subgroup analyses indicated that antibiotics are important sources of heterogeneity.

**Conclusion:**

This meta-analysis supports the effectiveness of nonsurgical treatments for CsA-induced GO, and comprehensive pre and posttransplant oral care is recommended. Further studies are needed to determine the biological mechanisms and influence of each variable on the efficacy of nonsurgical treatments in reducing drug-induced GO.

## 1. Introduction

Drug-induced gingival overgrowth (DIGO) is an iatrogenic disease characterized by enlarged and inflamed gums and bleeding upon probing. Severe DIGO is often disfiguring and can interfere with speech and mastication.^[1]^ Plaque is an important initiating factor in the development of DIGO. The accumulation of plaque triggers an inflammatory response in the gingiva, leading to the proliferation and dense arrangement of collagen fibers. Additionally, massive plaque accumulation in gingival pockets may be a persistent source of transient bacteremia, which increases the risk of systemic infections in immunocompromised patients and leads to profound complications.^[2]^

DIGO is a common side effect associated with 3 major drugs: anticonvulsants, calcium channel blockers, and immunosuppressants, particularly cyclosporin A (CsA).^[3]^ CsA has proven highly effective in preventing graft rejection through its potent T-cell suppression, which enables it to be extensively utilized in organ transplantation and in managing a range of autoimmune diseases like rheumatoid arthritis. More importantly, CsA is recommended as a front-line treatment of Aplastic anemia.^[4,5]^

The prevalence of CsA-induced gingival overgrowth (GO) in adults is approximately 25% to 30%, while in children, it exceeds 70%.^[6]^ Although the molecular mechanism underlying DIGO is still incompletely understood, it is believed the process is associated with an inflammatory response. This inflammation promotes fibroblast proliferation, leading to increased cell growth and excessive collagen synthesis. Additionally, CsA may aggravate fibroblast proliferation and collagen synthesis while inhibiting the degradation of the extracellular matrix (ECM).^[7]^ Furthermore, there is a correlation between GO severity and plaque control.^[8]^ The effects of nonsurgical periodontal treatment on CsA-induced GO have not been widely examined, and few case reports have been published.^[9,10]^ The nonsurgical treatment mostly involves supra- and subgingival scaling and root planing, and gingivectomy, which help control the periodontal inflammation and improve the oral microenvironment by removing dental calculus and plaque.^[10]^ A previous study has shown that nonsurgical periodontal therapy reduces inflammation, the depth of periodontal pockets, systemic bacteremia, and postsurgical infections to minimize the severity of GO.^[11]^ The removal of plaque and calculus decreases inflammatory mediators that contribute to fibroblast proliferation and excessive ECM deposition. And its recurrence decreases by reducing the local inflammation.^[12]^ However, a surgical approach has been suggested as the main choice for treating GO.^[12]^ Nevertheless, the high recurrence rate of GO following surgical therapy in patients was described by Ilgenli et al^[13]^ and can occur within 18 months, regardless of the drugs used to treat the condition.^[9]^

In this context, we conducted a meta-analysis to assess the effectiveness of nonsurgical periodontal treatment for patients with GO induced by CsA. Through our research, we hope that clinical workers can pay more attention to nonsurgical periodontal treatment and offer more alternatives to patients who are either unwilling or unable to accept the surgical treatment.

## 2. Materials and methods

This systematic review was conducted following the Cochrane Handbook for Systematic Review of Interventions and adhered to the Preferred Reporting Items for Systematic Reviews and Meta-Analyses (PRISMA) statement^[14]^ (Table S1, Supplemental Digital Content, https://links.lww.com/MD/P438). The systematic review protocol was registered in International Prospective Register of Systematic Reviews with the ID CRD42024570280. This paper conducted 2 separate meta-analyses to evaluate the efficacy of nonsurgical periodontal therapy in CsA-induced GO. Review One analyzed the differences of the final results between the nonsurgical periodontal treatment group and the control group without any periodontal therapy. Review Two conducted single groups of patients and analyzed the different outcome indicators before and after treatment (pretreatment vs posttreatment).

### 2.1. Search strategy

Development of the article retrieval strategy and acquisition of articles were performed by SL and YZ. Articles were searched from 5 databases (Cochrane Library, PubMed, EMBASE, China National Knowledge Infrastructure, and Chinese Academic Database). Supplementary retrievals from the Clinical Trials Database (clinicaltrials.gov) and Grey Literature Database (opengrey.eu) were also performed. Articles were retrieved using Medical Subject headings terms and subject terms: (“Cyclosporine” OR “Cyclosporin” OR “Ciclosporin” OR “CyclosporineA” OR “CyclosporinA” OR “Cyspin” OR “Neoral” OR “SandimmunNeoral” OR “CyA-NOF” OR “CYANOF” OR “Sandimmune” OR “Sandimmu” OR “CsA-Neoral” OR “CsA Neoral” OR “CsANeoral” OR “OL 27400” OR “OL27400”) AND (“Gingival Overgrowths” OR “Overgrowth, Gingival” OR “Overgrowths, Gingival” OR “Overgrowth Gingival” OR “Gingival Hyperplasias” OR “Hyperplasia, Gingival” OR “Gingival Hypertrophies”). The specific retrieval parameters are listed in Table S2, Supplemental Digital Content, https://links.lww.com/MD/P440. Additionally, references to the included studies were screened to make the search as comprehensive as possible. Some corresponding authors were contacted via e-mail for relevant information if the full text or key data could not be obtained. Articles were collected from the inception of each database to November 5th, 2024.

### 2.2. Article selection

Two authors (JB and XM) conducted a double-blind inclusion and exclusion of articles; a senior reviewer (SL) was consulted to resolve any disputes. Two authors browsed the article title and abstract as a preliminary screening step. The authors read the full text and determined the articles to be included in accordance with the Population, Intervention, Comparison, Outcomes and Study framework.^[15]^ The inclusion criteria were as follows:

(1) (P) Population: patients with GO caused by cyclosporine.
(2) (I) Intervention: nonsurgical periodontal treatment, including oral hygiene guidance, supra- and subgingival scaling, root planing, and supportive periodontal therapy.
(3) (C) Comparison: patients with DIGO who were untreated or only received periodontal oral health education.
(4) (O) Outcomes: primary outcomes included GO score and hypertrophy index (HI) (%), and secondary outcomes were probing depth (PD) (mm), probing depth percentage (PD %), plaque index percentage (%), and plaque index score.
(5) (S) Study: the article reported an interventional controlled study, such as a randomized controlled trial (RCT) or pre–post study.

The exclusion criteria were as follows: (1) population: patients taking other drugs with GO effects, such as calcium channel blockers; (2) intervention: dental surgery as an intervention; treatment with medication only; cyclosporine was stopped during periodontal treatment; (3) lack of key information or data after contacting the author; and (4) repeated articles from the same group of cases.

### 2.3. Data extraction

Data was extracted independently from the raw data provided in the original studies by the 2 reviewers (YR and JH), and divergences were resolved through third-party discussions (XY).

The following data were collected:

(1) Basic information on the studies: first author’s name, year of publication, and country.
(2) Characteristics of the study populations: age, sample size, sex, and basic diseases.
(3) Oral treatments were administered to the patients.
(4) Indicators reflecting periodontal conditions.

Owing to the limited number of studies and the variability in outcome indicators across different studies, the primary outcomes were as follows: (1) GO (score): the degree of GO was numerically graded on plaster study models of patients using the scoring method described by Seymour et al^[8]^ and (2) HI (%): interdental papillae were examined as described by Seymour et al giving a potential maximum score of 100 expressed as a percentage.^[8]^ The secondary outcomes were as follows: (3) PD (mm): recorded using a Williams 0 probe (Hu-Friedy, Chicago) at 6 points around each tooth; (4) PD (%): the number of sites that were at least 3 mm in depth was recorded and expressed as a percentage of the total number of sites measured, as reported by Loe and Silness^[16]^; (5) plaque index (PI) (%): the presence of plaque at the cervical area of all 4 tooth surfaces, expressed as a percentage^[17]^; and (6) PI (index): lingual, labial, and interproximal surfaces of the 12 anterior teeth were scored.^[18]^

### 2.4. Quality assessment

To evaluate the quality and risk bias of the included articles with the Effective Public Health Practice Project quality assessment tool, 2 researchers (JB and XM) conducted double-blind scoring, and controversies were resolved via third-party discussion (YR). This tool was deemed suitable for evaluating the methodological quality of various study types and designs.^[19]^ The scale evaluates articles in 6 dimensions: selection bias, study design, confounders, blinding, data collection methods, and withdrawals and drop-outs, with a rating of weak, moderate, or strong for each question. The global rating of each article follows the following criteria: no weak ratings in the 6 dimensions is “strong,” 1 weak rating in the 6 dimensions is “moderate,” and 2 or more weak ratings in the 6 dimensions is “weak.”

### 2.5. Statistical analysis

This meta-analysis was performed by RevMan 5.3 and Stata 12 (Stata Corporation, College Station). The results were reported as the standardized mean difference (SMD) and 95% confidence intervals (CI) to explore the effectiveness of nonsurgical periodontal treatments. Before the analysis, data presented as the median (interquartile range) were converted to the mean difference ± standard deviation.^[20]^ Cochran test and the I^2^ test were used to assess heterogeneity. If there was significant heterogeneity (*P *< .1), a random-effect model was selected. In contrast, for results with low heterogeneity (*P* ≥ .1), a fixed-effect model was selected.^[21]^ For results with high heterogeneity, subgroup analysis sorted by the area, sex, and antibiotics use was performed to identify the source of heterogeneity. Egger and Begg tests were used to evaluate publication bias.^[22]^ In cases with publication bias, the authenticity of the results was reanalyzed with the trim and fill method. The stability and reliability of the combined results were tested using sensitivity analysis (excluding studies individually).

## 3. Result

### 3.1. Article selection

**Figure 1.**
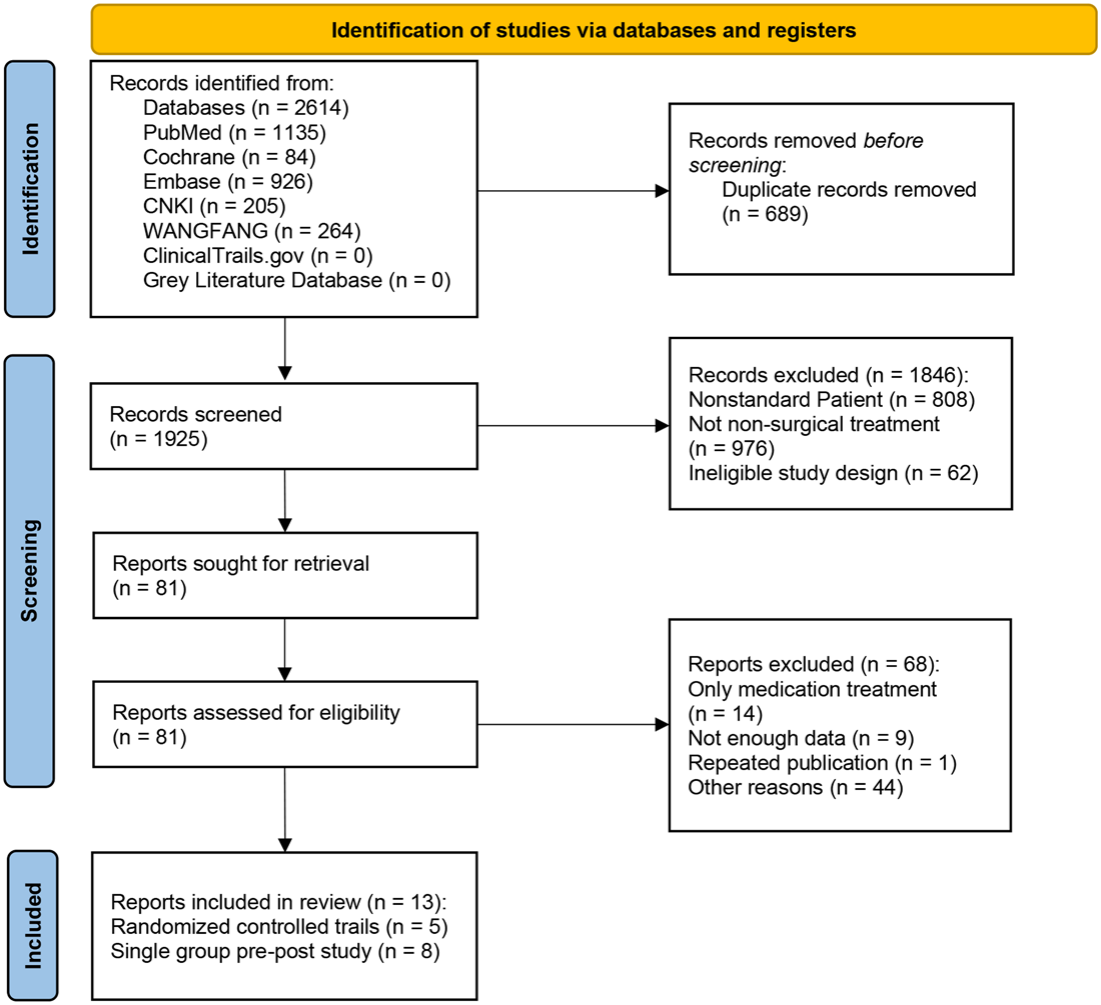
PRISMA 2020 flow diagram of research selection process.

Based on the PRISMA Guidelines, 2 researchers searched 7 databases (Cochrane Library, PubMed, EMBASE, China National Knowledge Infrastructure, Chinese Academic Database, Clinical Trials Database, and Grey Literature Database), and 2614 articles were retrieved. After excluding duplicate articles, 1925 articles were screened. According to the inclusion and exclusion criteria, 1844 articles were excluded. The specific reasons for exclusion and the process are presented in the PRISMA flow diagram (Fig. 1). Of the 13 articles finally included, there were 8 pre–post studies^[9,11,23–28]^ and 5 RCTs.^[8,29–32]^ The studies were conducted in Italy, China, Turkey, Britain, Morocco, Spain, and Iran.

Three RCTs^[8,30,31]^ were included in Review One, a meta-analysis that explored the efficacy differences between the nonsurgical periodontal treatment group and the control group. The other 2 RCTs were excluded because they did not include a blank control group^[32]^ or used nonstandard expression methods of the main outcome indicator (GO).^[29]^ There were 43 subjects in 3 RCTs, including 35 males and 8 females. Of the 49 control subjects, 40 were males and 9 were females. The mean age of the treatment groups was 34.13 to 46.3 years. During treatment, 16 subjects used antibiotics, whereas the other 27 did not. Detailed information of the patients described in the 3 articles is presented in Table 1.

**Table 1:** Characteristics of 3 RCTs (treatment group vs control group) included in meta-analysis.

Author	Country	Period	Treatment group						Control group					
			Sex(F/M	Age	HI (%)		PI (index)		Sex(F/M)	Age	HI (%)		PI (index)	
					Baseline	Final	Baseline	Final			Baseline	Final	Baseline	Final
Seymour et al^[8]^	UK	6 m	10/2	42.00 ± 11.00	10.60± 5.90	21.60± 14.60	0.84± 0.21	0.58± 0.26	13/2	41.00 ± 11.00	11.40± 6.20	29.80± 14.30	0.94± 0.30	1.33± 0.56
Kantarci et al^[30]^	Turkey	8 w	13/2	34.13 ± 13.07	53.40± 12.26	32.12± 11.99	1.30± 0.53	0.69± 0.25	13/3	33.31 ± 16.15	56.63± 8.88	52.83± 13.47	1.21± 0.54	1.15± 0.33
Gong et al^[31]^	China	6 m	12/4	46.30 ± 10.00	41.10± 8.60	30.70± 11.10	1.64± 0.52	1.25± 0.50	14/4	48.80 ± 9.30	46.20± 9.70	44.70± 9.70	1.48± 0.58	1.33± 0.55

Data are presented by mean and standard deviation.

F = female, HI = hypertrophy index, M = male, m = month, PI = plaque index, w = week.

The 13 articles^[8,9,11,23–32]^ were included in Review Two, which analyzed the differences of outcome indicators before and after intervention. It comprised 273 cases, and the average age of the participants was 21 to 58 years. Of the 273 cases, 199 used antibiotics during treatment. Specific information of the cases is presented in Table 2.

**Table 2:** Characteristics of pre–post studies (pretreatment group vs posttreatment group) included in meta-analysis.

Author	Country	Number of patients (M/F)	Age (year)	Follow-up period (month)	Antibiotic (yes/no)	GO		PD		PI	
						Baseline	Final	Baseline	Final	Baseline	Final
Aimetti et al^[13]^	Italy	21 (16/5)	51.95 ± 13.19	12	Yes (amoxicillin)	2.38 ± 1.92*	0.56 ± 0.83*	3.9 ± 1.40‡	2.1 ± 0.86‡	/	/
Aimetti et al^[24]^	Italy	21 (14/7)	46.05 ± 9.15	12	No	4.42 ± 0.24*	0.48 ± 0.68*	/	/	1.9 ± 0.56¶	0.33 ± 0.37¶
Castronovo et al^[11]^	Italy	44 (30/14)	55.05 ± 12.87	12	Yes (amoxicillin)	24.30 ± 24.91†	14.43 ± 18.78†	23.38 ± 27.14§	13.37 ± 14.38§	41.61 ± 28.61∥	35.45 ± 33.53∥
Gong et al^[31]^	China	16 (12/4)	46.3 ± 10	6	Yes (cefaclor)	41.40 ± 8.60†	30.70 ± 11.10†	3.44 ± 0.85‡	2.62 ± 0.96‡	1.64 ± 0.52¶	1.25 ± 0.50¶
Gong et al^[32]^	China	15 (7/8)	44.8 ± 9.1	1	Yes (cefaclor + roxithromycin)	42.54 ± 17.18†	19.90 ± 9.81†	3.38 ± 0.79‡	2.2 ± 0.47‡	1.39 ± 0.89¶	0.69 ± 0.55¶
Iudicibus et al^[26]^	Italy	45 (35/10)	49.2 (26–75)	6	Yes (amoxicillin/clarithromycin)	30.79 ± 3.40†	16.57 ± 1.80†	33.42 ± 4.3§	17.41 ± 2.5§	49.8 ± 4.1∥	30.53 ± 3.40∥
Kantarci et al^[30]^	Turkey	15 (13/2)	34.13 ± 13.07	2	No	53.40 ± 12.26†	32.13 ± 11.99†	3.75 ± 1.20‡	2.97 ± 0.8‡	1.30 ± 0.53¶	0.69 ± 0.25¶
Li et al^[27]^	China	12 (8/4)	34.50 ± 5.20	6	No	/	/	5.03 ± 0.51‡	2.25 ± 0.27‡	1.54 ± 0.42¶	0.48 ± 0.18¶
Malek et al^[9]^	Morocco	1 (0/1)	21	24	Yes (amoxicillin + clavulanic acid)	30.50†	0	/	/	/	/
Romano et al^[28]^	Italy	1 (1/0)	58	12	No	3.20 ± 1.5*	0.3 ± 0.5*	8.1 ± 2.90‡	4.2 ± 1.50‡	/	/
Seymour et al^[8]^	UK	12 (10/2)	42 ± 11	6	No	10.60 ± 5.90†	21.60 ± 14.60†	0.25 ± 12.56§	11.6 ± 16.40§	0.835 ± 0.28¶	0.584 ± 0.26¶
Shi et al^[25]^	China	57 (38/19)	34.5	6	Yes (metronidazole)	/	/	3.03 ± 1.08‡	2.91 ± 1.14‡	2.97 ± 1.31¶	1.91 ± 0.77¶
Somacarrera et al^[29]^#	Spain	13	/	6	No	4.24 ± 1.37 (mm)#	3.47 ± 0.95 (mm)#	/	/	62.76 ± 5.44∥	33.53 ± 7.66∥

Data is presented by mean and standard deviation.

F = female, GO = gingival overgrowth, M = male, PD = probing depth, PI = plaque index.

* Gingival overgrowth (score).

† Hypertrophy index (%).

‡ Probing depth (mm).

§ Probing depth (%).

∥ Plaque index (%).

¶ Plaque index (index).

# The GO score in this study cannot be converted into a percentage, thus it cannot be analyzed.

### 3.2. Assessment of risk of bias

The evaluation results of the included articles using the Effective Public Health Practice Project scale are shown in Table 3. Two articles are rated as strong,^[29,32]^ 7 articles are rated as moderate,^[8,11,23–25,27,31]^ and 4 articles are rated as weak.^[9,26,28,30]^

**Table 3:** Quality assessment of all included articles by Effective Public Heath Practice Project (EPHPP) quality assessment tool.

Author	Selection bias	Study design	Confounders	Blinding	Data collection methods	Withdrawals and drop-outs	Overall rating
Aimetti et al^[13]^	Strong	Moderate	Strong	Weak	Strong	Strong	Moderate
Aimetti et al^[24]^	Strong	Moderate	Strong	Weak	Strong	Strong	Moderate
Castronovo et al^[11]^	Strong	Moderate	Strong	Weak	Strong	Moderate	Moderate
Gong et al^[31]^	Strong	Strong	Strong	Weak	Strong	Strong	Moderate
Gong et al^[32]^	Strong	Strong	Strong	Strong	Strong	Strong	Strong
Iudicibus et al^[26]^	Strong	Moderate	Strong	Weak	Strong	Weak	Weak
Kantarci et al^[30]^	Strong	Strong	Strong	Weak	Strong	Weak	Weak
Li et al^[27]^	Strong	Moderate	Strong	Weak	Strong	Strong	Moderate
Malek et al^[9]^	Strong	Weak	Strong	Weak	Strong	Strong	Weak
Romano et al^[28]^	Strong	Weak	Strong	Weak	Strong	Strong	Weak
Seymour et al^[8]^	Strong	Strong	Strong	Weak	Strong	Strong	Moderate
Shi et al^[25]^	Strong	Moderate	Strong	Weak	Strong	Strong	Moderate
Somacarrera et al^[29]^	Strong	Strong	Strong	Strong	Strong	Strong	Strong

Strong overall rating = 0 weak ratings; moderate overall rating = 1 weak ratings; weak overall rating = 2 or more weak ratings.

### 3.3. Meta-analysis of Review One

#### 3.3.1. Consolidated results of HI (%)

**Figure 2.**
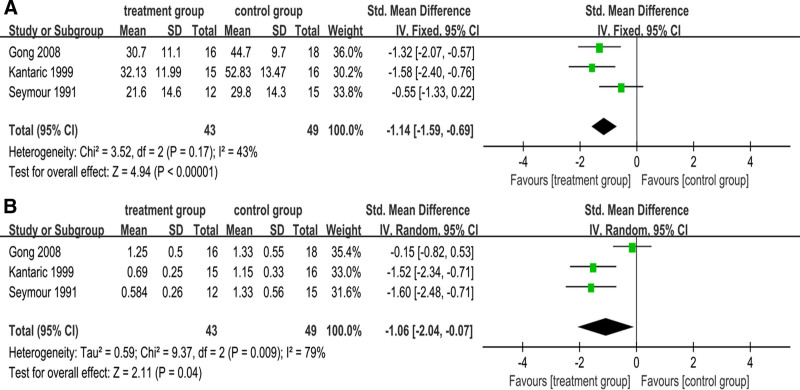
(A) Meta-analysis of influence of nonsurgical periodontal therapy on HI % (treatment group vs control group). (B) Meta-analysis of influence of nonsurgical periodontal therapy on PI index (treatment group vs control group).

The results of pooled meta-analysis of the 3 studies^[8,30,31]^ are shown in Figure 2A. Because of the low heterogeneity in the statistical results (I^2^ = 43%, *P* = .17), the fixed-effect model was used to analyze the statistical outcomes. After nonsurgical treatment, the HI (%) in the treatment group was significantly lower than that in the control group (SMD = −1.14; 95% CI: [−1.59, −0.69]).

#### 3.3.2. Consolidated results of PI (index)

Because of the high heterogeneity of the 3 studies^[8,30,31]^ (I^2^ = 79%, *P* = .009), the random-effect model was applied. The PI (index) of the treatment group was significantly lower than that of the control group (SMD = −1.06; 95% CI: [−2.04, −0.07]) (see Fig. 2B). A subgroup analysis was performed to explore the sources of the high heterogeneity further.

### 3.4. Meta-analysis of Review Two

The 6 analyzed factors (GO (score), HI (%), PD (mm), PD (%), PI (%), and PI (index)) were evaluated using a random-effect model due to high heterogeneity (Figure S1, Supplemental Digital Content, https://links.lww.com/MD/P442). The pooled results showed that 3 outcome indicators (HI (%), PD (mm), and PI (index)) significantly decreased after nonsurgical treatment. The other 3 indicators (GO (score), PD (%), and PI (%)) did not significantly differ before and after treatment. Specific information on each outcome indicator in the meta-analysis, including the number of studies, heterogeneity, and data of consolidated results, are summarized in Table S3, Supplemental Digital Content, https://links.lww.com/MD/P441. The high heterogeneity of the results suggests that further subgroup analysis is required.

### 3.5. Subgroup analysis

The results of subgroup analyses divided by area are presented in Table S4, Supplemental Digital Content, https://links.lww.com/MD/P441 and Figure S2, Supplemental Digital Content, https://links.lww.com/MD/P442. The results showed that the PI (index) did not significantly differ between the treatment and control groups in the Asian population (SMD = −0.82; 95% CI: [−2.16, 0.53]), whereas the European population showed significant differences (SMD = −1.60 [−2.48, −0.71]). In contrast, there was no significant difference in the HI (%) and PI (index) before and after treatment in the European population, whereas treatment was significantly effective in the Asian population.

Studies were also categorized according to the male-to-female ratio (M/F > 1 and M/F < 1) (see Table S5, Supplemental Digital Content, https://links.lww.com/MD/P441 and Figure S3, Supplemental Digital Content, https://links.lww.com/MD/P442). The results for each group showed significant heterogeneity. The HI (%) in the group with M/F > 1 showed no significant difference before and after treatment; however, in the group with M/F < 1, this index decreased significantly.

Regarding antibiotic use, there was no significant difference in the PI (index) between the treated and untreated groups (SMD = −0.15; 95% CI: [−0.82, 0.53]). However, in the subgroup without antibiotics, the PI (index) of the test groups was significantly lower than that in the control groups and showed low heterogeneity (SMD = −1.56; 95% CI: [−2.16, −0.96], I^2^ = 0, *P* = .91). In contrast, HI (%) and PD (mm) decreased significantly after treatment with antibiotic based on pre and posttreatment comparisons (HI (%) (SMD = −2.04; 95% CI: [−3.95, −0.14]), PD (mm) (SMD = −1.02; 95% CI: [−1.87, −0.18])), while no significant difference was observed before and after treatment in studies without antibiotics (HI (%) (SMD = −0.38; 95% CI: [−2.98, 2.23], PD (mm) (SMD = −3.57; 95% CI: [−9.28, 2.15])). The specific analysis data are summarized in Table S6, Supplemental Digital Content, https://links.lww.com/MD/P441 and illustrated in Figure S4, Supplemental Digital Content, https://links.lww.com/MD/P442.

### 3.6. Publication bias

In Egger test, the PD (mm) showed some publication bias (*P* = .003). The PI (index) of the meta-analysis in pre- and poststudies showed publication bias in Begg test (*P* = .016). No significant publication bias was observed in other outcome indicators in either Egger test or Begg test (*P* > .05). The results are listed in Table S7, Supplemental Digital Content, https://links.lww.com/MD/P441 and shown in Figure S5, Supplemental Digital Content, https://links.lww.com/MD/P442. The combined effects of PD (mm) and PI (index) were reanalyzed using the trim and filling method, and different results were obtained (PD (mm): SMD = 0.086 [0.018, 0.406]; PI (index): SMD = 0.207; 95% CI: [0.106, 0.404]).

### 3.7. Sensitivity analysis

The results of the sensitivity analysis of 3 RCTs revealed no significant difference in the PI (index) between the treated and untreated groups after removing the studies by Kantarci et al^[30]^ and Seymour et al.^[8]^ The results of the sensitivity analysis of the HI (%) showed good stability. Sensitivity analysis with the HI (%) and GO (score) of the pre- and posttreatment in the included studies showed different results. After removing the study by Castronovo et al,^[11]^ the results of the PI (%) changed from showing a difference to showing no significant difference. The statistical results for the PD (mm), PD (%), and PI (index) were stable in the sensitivity analysis.

## 4. Discussion

CsA-induced GO is often observed in patients who have undergone organ transplants. This meta-analysis was performed because few evidence-based studies have focused on the efficacy of nonsurgical periodontal therapy for treating CsA-induced GO. Current evidence indicates that nonsurgical periodontal therapy improves cyclosporine-induced GO. We found that antibiotic use may affect the efficacy of this treatment in improving HI (%) and PD (mm) after treatment, as antibiotics were primarily employed to prevent potential infections following periodontal therapy. However, our results should be interpreted with caution because of the methodological shortcomings and substantial heterogeneity among the included studies.

CsA-induced GO involves fibrotic enlargement of the CsA origin and inflammatory lesions caused by microbial dental plaque.^[30]^ Clinical inflammation can be controlled through strict nonsurgical periodontal treatment and results in gingival tissue that is histologically and immunohistochemically comparable to that of healthy control patients.^[24]^ Many studies have shown that nonsurgical treatment is effective for GO caused by cyclosporine, but no relevant review has been published. To explore the outcomes of clinical treatment for GO, this review performed a meta-analysis (treatment vs control groups and pretreatment vs post-treatment groups) based on the GO (score), HI (%), PD (mm), PD (%), PI (%), and PI (index) as outcomes. Three outcome indicators (HI (%), PD (mm), and PI (index)) were significantly decreased after nonsurgical treatment, while no significant changes were observed in GO (score), PD (%), and PI (%). Montebugnoli et al^[33]^ reported that gingival volume changes much more slowly than plaque levels after nonsurgical treatment. The slow pace of gingival volume changes in CsA-induced GO (GO) and the subsequent lack of significant improvement in the GO (score) may be attributed to the fact that the accumulated ECM requires a considerable amount of time to be cleared, particularly after inflammation has been controlled. Since the changes in these periodontal outcomes are highly time-dependent, a significant source of variability in these indicators may be the differences in follow-up periods reported across studies, ranging from 1 to 24 months.^[9,32]^ Also, baseline data may have an impact on such trend discrepancy, as patients in part studies already had periodontal indexes as PD (mm) shown in Table 2 close to normal levels before treatment, which likely resulted in minimal change after periodontal therapy. Seymour et al^[8]^ reported that after periodontal therapy, some patients’ PD increased, which challenges the notion that nonsurgical periodontal therapy and oral hygiene programs consistently improve the GO. This finding suggests significant challenges related to patient compliance and the quality of treatment administration.^[8]^ Further analyses based on treatment protocols, baseline periodontal status, and the length of follow-up are needed to eliminate the interference of the above factors.

Considering the high heterogeneity of the results, subgroup analyses (i.e., area, gender, and antibiotics) were also performed. We found that there were only 9 females (of 49 control subjects), and in the 3 RCTs, only 8 of the 43 subjects were female. Sex hormones, like testosterone, were reported to be a modifying factor in periodontal disease pathogenesis, as they can enhance the gingival inflammatory response to dental plaque and influence the severity of GO.^[34]^ However, many cyclosporin studies have a significant male bias since organ transplantation is more frequently carried out on males,^[35]^ raising questions about the role of sex hormones in these cases. Thomason et al used stepwise regression techniques to analyze the effects of a range of risk factors, while Ellis et al found that males are 3 times more likely to get GO than females with calcium channel blockers.^[35]^ Both studies reported that males were at greater risk of developing this unwanted effect than females, but the relevant effects of sex hormones are still unclear. In the subgroup analysis of European and Asian populations, significant changes in HI (%) and PI (Index) were observed in the Asian group but not in the European group after treatment. This could be related to Seymour et al report,^[8]^ which showed an increase in HI (%) rather than a decrease in Europeans. Additionally, higher heterogeneity in European studies may have influenced the changes in these indicators. Antibiotics may be a critical indicator of the effectiveness of nonsurgical periodontal treatments. Although there is no evidence-based research or guidelines for this prediction, prophylactic antibiotics are generally recommended. In the studies that used antibiotics,^[9,11,23–27,30,32]^ these antibiotics were applied as a measure to prevent infection after periodontal therapy. Moreover, 2 studies^[25,32]^ used antibiotics as an adjunct to nonsurgical therapy. The use of antibiotics in routine nonsurgical treatment varies, including the indications, dosage, and treatment time. In the subgroup analysis of the 3 RCTs, the PI (index) did not significantly differ between the treatment and control groups with antibiotics. Gong et al^[31]^ observed no significant difference in the PI (index) between the treated and untreated groups at 1 and 6 months after nonsurgical periodontal treatment, whereas a remarkable difference was observed at 3 months. Considering that all patients received oral hygiene instructions at the beginning of therapy, effective home plaque control may explain why the control groups had significantly lower PI values. However, the GO (score) and PD (mm) remarkably differed at 1, 3, and 6 months after periodontal treatment, indicating a significant benefit of this treatment. Seymour et al^[8]^ and Gong et al^[32]^ reported that patients who received nonsurgical periodontal therapy were administered antibiotics (0.25 g cefaclor, 1 hour preoperatively). This result should be considered with caution, as the PI (index) showed no significant differences between subgroups with and without antibiotics. Gong et al^[32]^ reported that patients were administered 300 mg of roxithromycin once daily for 5 days, and Shi et al^[25]^ reported on patients treated with 200 mg of metronidazole thrice daily for 7 days after initial periodontal treatment. However, the study result of GO (score) showed significant differences between the subgroups with and without antibiotics in the meta-analysis (pre- vs posttreatment). Previous studies have reported that the combination of azithromycin, roxithromycin, or metronidazole with nonsurgical therapy improves the GO status and shows anti-inflammatory and immunomodulatory effects on CsA-induced GO.^[32,36,37]^ As shown in Table 2, amoxicillin used as a preventive measure had little impact on GO and PD, and metronidazole showed no significant improvement in PD. However, the combination therapy of roxithromycin and cefaclor resulted in greater improvements in both GO and PD compared to cefaclor alone as a preventive measure. There were significant differences between subgroups according to the specific types of antibiotics. However, the included studies had inconsistent inclusion criteria and drug usage patterns; thus, these results should be further verified. In addition to organ transplants, systemic conditions include hypertension and pustular psoriasis.^[28]^ Calcium channel blockers may worsen the situation in patients with hypertension and CsA-induced GO. Considering the severity of CsA-induced GO in various studies, this may be 1 reason for the instability in the results of treatment outcomes. Aimetti et al^[23,24]^ evaluated CsA-treated patients who had severe overgrowth (>30%) and did not have alveolar bone loss. Overall, prophylactic antibiotic protocols should be established in consultation with the patient’s doctor.^[38]^ Significant variations were observed in the indicators of the periodontal condition. In addition to the 6 primary outcomes, other supplementary results were in the included articles. The bleeding index was calculated as the percentage of sites exhibiting bleeding at the gingival margin upon probing. De Iudicibus et al^[26]^ found that the bleeding index was significantly decreased at 6 months after starting nonsurgical periodontal therapy as their risk of bias was rated as weak. Aimetti et al^[23]^ determined the full-mouth plaque score and full-mouth bleeding score and observed 42% and 34% decreases, respectively, in these values at 12 months after treatment. These indicators can reflect partial clinical changes of the CsA-induced GO, but the changes may be inconsistent with results of 6 primary outcomes. Kantarci et al reported that the calculus index in the test group with periodontal treatment did not significantly differ from the controls without periodontal treatment when PD and GO showed significant improvement during following therapy.^[30]^ However, it should be noted that their research exhibited a high risk of bias.

The correlation between the CsA dosage and GO development is controversial.^[39]^ Various studies have suggested that pharmacokinetic variables of immunosuppressive therapy, such as the doses and serum levels of agents, can be regarded as risk markers for the severity of overgrowth.^[40–44]^ Threshold levels may vary among individuals because of cyclosporine and dose, plasma concentrations of cyclosporine, and preexisting conditions of GO. A certain threshold level of cyclosporine is required to induce gingival changes, considering that its blood level is unstable.^[30]^ The specific usage of clinical drugs should be further studied in a large patient population.^[45]^

Individual susceptibility may be associated with a genetic predisposition that influences fibroblastic heterogeneity.^[11,26]^ Preliminary studies have suggested the C3435T polymorphism as a genetic factor potentially influencing the course of CsA-induced GO in transplant patients subjected to periodontal therapy.^[26]^

### 4.1. Strengths and limitations of this study

This review explored the effectiveness of the nonsurgical treatment of GO through meta-analysis and provided a feasible treatment plan for GO caused by drugs in the future, make more dentists choose nonsurgical periodontal therapy rather than surgical therapy first. It used strict eligibility criteria and included comparable randomized studies, which contributed to the reliability of the results. This article also analyzed the sources of heterogeneity of results from different aspects and made a reasonable discussion on the impact of antibiotics on treatment. However, this study had some limitations. First, because the information reported in the included literature was inconsistent, a more comprehensive subgroup analysis could not be performed. Furthermore, this review did not stratify the patients according to the cyclosporine dose or underlying diseases. Sensitivity analysis was performed because of the varying quality of individual studies, the results showed little difference compared to those of before risk-based analysis. However, some data were reported without adjusting for potential confounders; thus, the pooled results may be prone to bias. Various outcome measures have been reported for GO reduction. Limited data on the same indicators make it difficult to draw reliable conclusions. To more precisely identify the prognostic factors of nonsurgical treatment in treated patients with GO, a multicenter study should be performed to strengthen the clinical evidence by including more variables.

Thus, it would be relevant to inquire about the influence of control measures on CsA-induced GO and the degree of the patients’ condition. In the future, it is necessary to explore the impact of control measures on CsA-induced GO and the correlation between the efficacy and the baseline severity of the patient’s condition, and specifically, if the different types of antibiotics used can modify the risk; to investigate whether it is affected by cyclosporine dose, underlying diseases, or GO values at baseline; to determine which clinical parameters and biomedical index (full-mouth bleeding score,^[23]^ calculus index (CI),^[30]^ blood level of cyclosporine, etc) could behave as reliable markers.

## 5. Conclusion

The results of this systematic review reinforce and complement the evidence regarding the efficacy of nonsurgical treatment in reducing GO induced by CsA. Regardless of the drug regimen, adequate pre- and posttransplant oral healthcare is recommended. Further studies are needed to determine the biological mechanisms, the influence of factors such as follow-up duration and antibiotic therapy on treatment outcomes, and the role of each variable in the efficacy of periodontal debridement in reducing GO. In addition, the critical GO score that requires nonsurgical treatment should be evaluated in accordance with individual functional and aesthetic requirements.

## Author contributions

**Conceptualization:** Shan Liu, Xinxin Lv.

**Data curation:** Jiaan Hu, Yuting Ruan, Xue Yang.

**Formal analysis:** Jiaan Hu, Yuting Ruan, Nanzhen Lin, Xinyi Mao.

**Investigation:** Jiaan Hu, Yuting Ruan, Nanzhen Lin, Ying Zhang, Xinyi Mao, Junyi Bai.

**Methodology:** Ying Zhang, Shan Liu, Xinxin Lv.

**Project administration:** Shan Liu, Xinxin Lv

**Supervision:** Shan Liu, Xinxin Lv

**Validation:** Jiaan Hu, Yuting Ruan, Nanzhen Lin, Shan Liu, Xinxin Lv.

**Writing – original draft:** Jiaan Hu, Yuting Ruan, Shan Liu, Xinxin Lv.

**Writing – review & editing:** Jiaan Hu, Yuting Ruan, Nanzhen Lin, Xinyi Mao, Junyi Bai, Shan Liu, Xinxin Lv.

## Supplementary Material


