# Supplementary material for: Non-surgical treatment of cyclosporin A-induced gingival overgrowth: A systematic review and meta-analysis
Source: Medicine (Baltimore). 2025 Jul 18;104(29):e43434. doi: 10.1097/MD.0000000000043434 (PMC12282823; doi:10.1097/MD.0000000000043434)
Supplement: Supplementary file 2 [file medi-104-e43434-s002.pdf]

Retrieval strategy

Databases	Search terms	Number of records
PubMed	#1 (((("Gingival Overgrowth"[Mesh Terms]) OR "Gingival Hyperplasia"[Mesh Terms]) OR "Gingival Hypertrophy"[Mesh Terms]) OR (gingival enlargement[MeSH Terms])) OR (gingival enlargements[MeSH Terms])<br>#2 ((gingival overgrowth[Title]) OR (Gingival Hyperplasia[Title])) OR (Gingival Hypertrophy[Title])<br>#3 (((((((((Gingival Overgrowths[Title]) OR (Overgrowth, Gingival[Title])) OR (Overgrowths, Gingival[Title])) OR (Overgrowth Gingival[Title])) OR (Gingival Hyperplasias[Title])) OR (Hyperplasia, Gingival[Title])) OR (Hyperplasias, Gingival[Title])) OR (Gingival Hypertrophies[Title])) OR (Hypertrophies, Gingival[Title])) OR (Hypertrophy, Gingival[Title])<br>#4 (gingival enlargements[Title]) OR (gingival enlargement[Title])<br>#5 #1 OR #2 OR #3 OR #4<br>#6 ((((((((((((((((((Cyclosporine[Title]) OR (Cyclosporin[Title])) OR (Ciclosporin[Title])) OR (Cyclosporine A[Title])) OR (Cyclosporin A[Title])) OR (Cyspin[Title])) OR (Neoral[Title])) OR (Sandimmun Neoral[Title])) OR (CyA-NOF[Title])) OR (CyA NOF[Title])) OR (Sandimmune[Title])) OR (Sandimmun[Title])) OR (CsA-Neoral[Title])) OR (CsA Neoral[Title])) OR (CsANeoral[Title])) OR (OL 27-400[Title])) OR (OL 27 400[Title])) OR (OL 27400[Title])) OR (medication-induced[Title])) OR (drug-induced[Title])<br>#7 Cyclosporine[MeSH Terms]<br>#8 Ciclosporin[MeSH Terms]<br>#9 #6 OR #7 OR #8<br>#10 #9 AND #5	1135
Embase	#1 'gingiva overgrowth'/exp/mj<br>#2 'gingiva hyperplasia'/exp/mj<br>#3 'gingiva hypertrophy'/exp/mj<br>#4 'gingival overgrowth':ti OR 'gingival hyperplasia':ti OR 'gingival hypertrophy':ti OR 'gingival hyperplasias':ti OR 'gingival hypertrophies':ti OR 'gingival enlargement':ti OR 'gingival enlargements':ti<br>#5 #1 or #2 or #3 or #4 or #5<br>#6 'cyclosporine'/exp/mj<br>#7 'cyclosporine':ti OR 'cyclosporin':ti OR 'ciclosporin':ti OR 'cyclosporine a':ti OR 'cyclosporin a':ti OR 'cyspin':ti OR 'neoral':ti OR 'sandimmun neoral':ti OR 'cya nof':ti OR 'sandimmune':ti OR 'sandimmun':ti OR 'csa neoral':ti OR 'csaneoral':ti OR 'ol 27-400':ti OR 'ol 27 400':ti OR 'ol 27400':ti OR 'medication-induced':ti OR 'drug-induced':ti<br>#8 #6 OR #7<br>#9 #5 AND #8	926
Cochrane	#1 MeSH descriptor: [Gingival Overgrowth] explode all trees	84

Library	#2 MeSH descriptor: [Gingival Hyperplasia] explode all trees<br>#3 MeSH descriptor: [Gingival Hypertrophy] explode all trees<br>#4 (gingival overgrowth or gingival Hyperplasia or gingival Hypertrophy):ti,ab,kw<br>#5 (Gingival Overgrowths or Overgrowth, Gingival or Overgrowths, Gingival or Overgrowth Gingival or Gingival Hyperplasias or Hyperplasia, Gingival or Hyperplasias, Gingival or Gingival Hypertrophies or Hypertrophies, Gingival or Hypertrophy, Gingival):ti,ab,kw<br>#6 (gingival enlargement or gingival enlargements):ti,ab,kw<br>#7 #1 OR #2 OR #3 OR #4 OR #5 OR #6<br>#8 ("Cyclosporine" OR "Cyclosporin" OR "Ciclosporin" OR "Cyclosporine A" OR "CyclosporinA" OR "Cyspin" OR "Neoral" OR "Sandimmun Neoral" OR "CyA-NOF" OR "CyA NOF" OR "Sandimmune" OR "Sandimmun" OR "CsA-Neoral" OR "CsA Neoral" OR "CsANeoral" OR "OL 27-400" OR "OL 27 400" OR "OL27400" OR "drug-induced" OR "medication-induced"):ti<br>#9 MeSH descriptor:[Cyclosporine]explodeall trees<br>#10 MeSH descriptor: [Cyclosporins] explode all trees<br>#11 #8 OR #9 OR #10<br>#12 #7 AND #11	
CNKI	SU=(环孢多肽 A+环孢灵+环孢霉素+赛斯平+山地明+环孢+新山地明+环孢素) AND SU=(牙龈过度生长+牙龈肥大+牙龈增生)	205
Wanfang	主题:(环孢多肽 or 环孢灵 or 环孢霉素 or 赛斯平 or 山地明 or 环孢 or 新山地明 or 环孢素) and 主题:(牙龈过度生长 or 牙龈肥大 or 牙龈增生)	264
