# Supplementary material for: Non-surgical treatment of cyclosporin A-induced gingival overgrowth: A systematic review and meta-analysis
Source: Medicine (Baltimore). 2025 Jul 18;104(29):e43434. doi: 10.1097/MD.0000000000043434 (PMC12282823; doi:10.1097/MD.0000000000043434)
Supplement: Supplementary file 3 [file medi-104-e43434-s003.pdf]

(a)

Study or Subgroup	post treatment Mean	SD	Total	pre treatment Mean	SD	Total	Weight	Std. Mean Difference IV, Random, 95% CI	Std. Mean Difference IV, Random, 95% CI
Aimetti 2005	0.56	0.83	21	2.38	1.92	21	50.9%	-1.21 [-1.87, -0.54]	
Aimetti 2008	0.48	0.68	21	4.24	0.24	21	49.1%	-7.23 [-8.97, -5.50]	
Romano 2015	0.3	0.5	1	3.2	1.5	1		Not estimable	
**Total (95% CI)**			**43**			**43**	**100.0%**	**-4.17 [-10.07, 1.74]**	

Heterogeneity: Tau² = 17.72; Chi² = 40.49, df = 1 (P < 0.00001); I² = 98%
Test for overall effect: Z = 1.38 (P = 0.17)

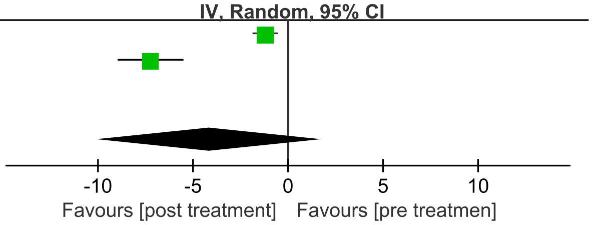

(b)

	post treatment			pre treatment				Std. Mean Difference
Study or Subgroup	Mean	SD	Total	Mean	SD	Total	Weight	IV, Random, 95% CI
Castronovo 2014	14.43	18.78	44	24.3	24.91	44	17.3%	-0.44 [-0.87, -0.02]
Gong 2008	30.7	11.1	16	41.4	8.6	16	16.7%	-1.05 [-1.80, -0.31]
Gong 2014	19.9	9.81	15	42.54	17.18	15	16.5%	-1.57 [-2.41, -0.74]
Iudicibus 2013	16.57	1.8	45	30.79	3.4	45	16.4%	-5.18 [-6.06, -4.31]
Kantarci 1999	32.13	11.99	15	53.4	12.26	15	16.5%	-1.71 [-2.56, -0.85]
Malek 2019	0	0	1	30.5	0	1		Not estimable
Seymour 1991	21.6	14.6	12	10.6	5.9	12	16.5%	0.95 [0.10, 1.81]
**Total (95% CI)**			**148**			**148**	**100.0%**	**-1.49 [-2.92, -0.06]**

Heterogeneity: $Tau^2 = 3.06$; $Chi^2 = 117.85$, $df = 5$ ($P < 0.00001$); $I^2 = 96\%$
Test for overall effect: $Z = 2.04$ ($P = 0.04$)

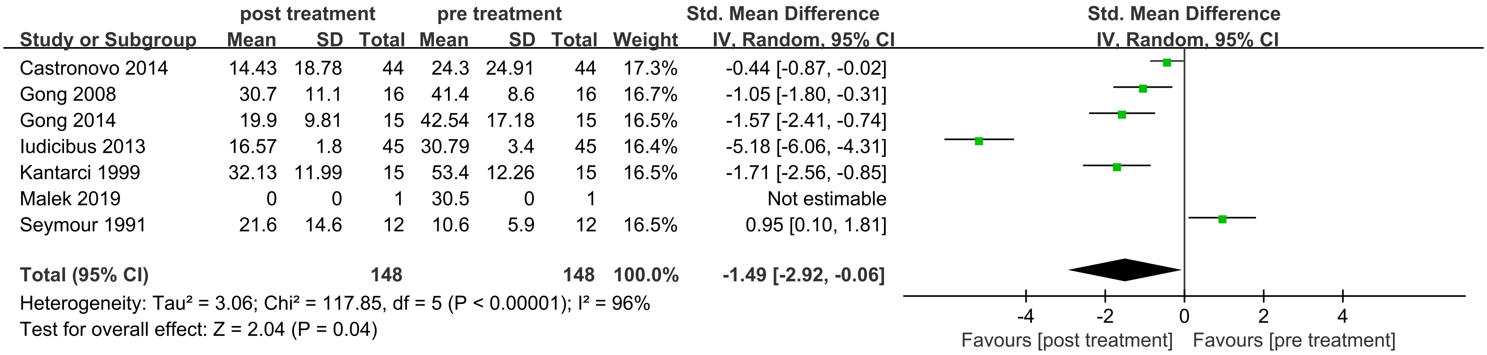

(c)

Study or Subgroup	post treatment Mean	SD	Total	pre treatment Mean	SD	Total	Weight	Std. Mean Difference IV, Random, 95% CI
Aimetti 2005	2.1	0.86	21	3.9	1.4	21	18.1%	-1.52 [-2.21, -0.83]
Gong 2008	2.62	0.96	16	3.44	0.85	16	17.9%	-0.88 [-1.61, -0.15]
Gong 2014	2.2	0.47	15	3.38	0.79	15	17.2%	-1.77 [-2.63, -0.90]
Kantarci 1999	2.97	0.8	15	3.75	1.2	15	17.8%	-0.74 [-1.49, -0.00]
Li 2013	2.25	0.27	12	5.03	0.51	12	9.6%	-6.58 [-8.77, -4.39]
Romano 2015	4.2	1.5	1	8.1	2.9	1		Not estimable
Shi 2008	2.91	1.14	57	3.03	1.08	57	19.5%	-0.11 [-0.47, 0.26]
**Total (95% CI)**			**137**			**137**	**100.0%**	**-1.52 [-2.46, -0.58]**

Heterogeneity: $Tau^2 = 1.14$; $Chi^2 = 49.19$, df = 5 ($P < 0.00001$); $I^2 = 90\%$
Test for overall effect: Z = 3.18 (P = 0.001)

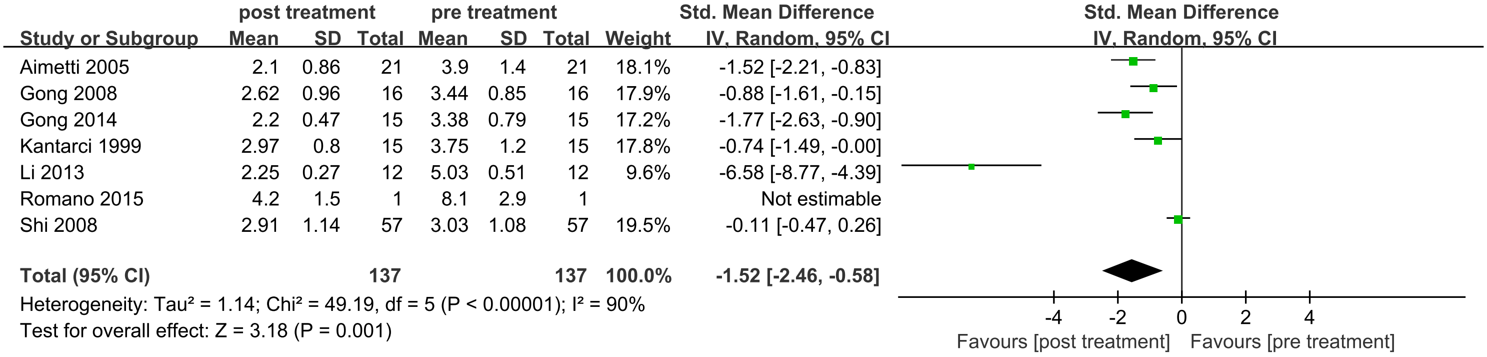

(d)

	post treatment			pre treatment				Std. Mean Difference
**Study or Subgroup**	**Mean**	**SD**	**Total**	**Mean**	**SD**	**Total**	**Weight**	**IV, Random, 95% CI**
Castronovo 2014	13.37	14.38	44	23.38	27.14	44	33.8%	-0.46 [-0.88, -0.03]
Iudicibus 2013	17.41	2.5	45	33.42	4.3	45	33.1%	-4.51 [-5.30, -3.72]
Seymour 1991	11.6	16.4	12	0.25	12.56	12	33.0%	0.75 [-0.08, 1.58]
**Total (95% CI)**			**101**			**101**	**100.0%**	**-1.40 [-4.12, 1.32]**

Heterogeneity: Tau² = 5.66; Chi² = 99.09, df = 2 (P < 0.00001); I² = 98%
Test for overall effect: Z = 1.01 (P = 0.31)

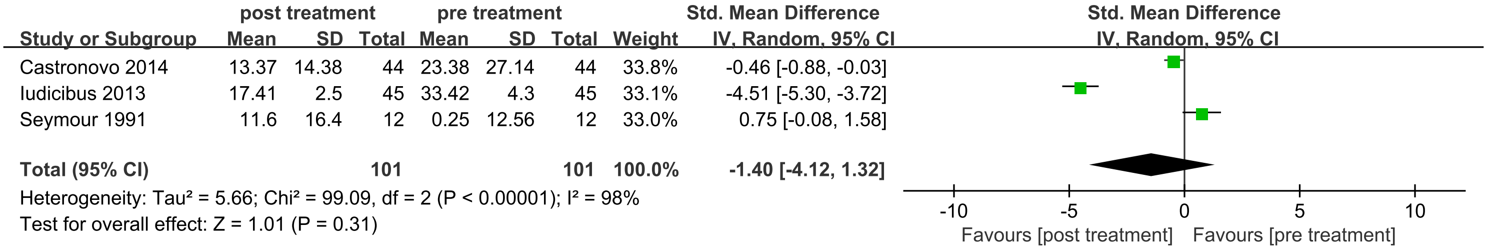

(e)

Study or Subgroup	post treatment Mean	SD	Total	pre treatment Mean	SD	Total	Weight	Std. Mean Difference IV, Random, 95% CI
Castronovo 2014	35.45	33.53	44	41.61	28.61	44	34.0%	-0.20 [-0.61, 0.22]
Iudicibus 2013	30.53	3.4	45	49.8	4.1	45	33.6%	-5.07 [-5.94, -4.21]
Somacarrera 1997	33.53	7.66	13	62.76	5.44	13	32.4%	-4.26 [-5.73, -2.79]
**Total (95% CI)**			**102**			**102**	**100.0%**	**-3.15 [-6.81, 0.51]**

Heterogeneity: Tau² = 10.18; Chi² = 115.57, df = 2 (P < 0.00001); I² = 98%
Test for overall effect: Z = 1.69 (P = 0.09)

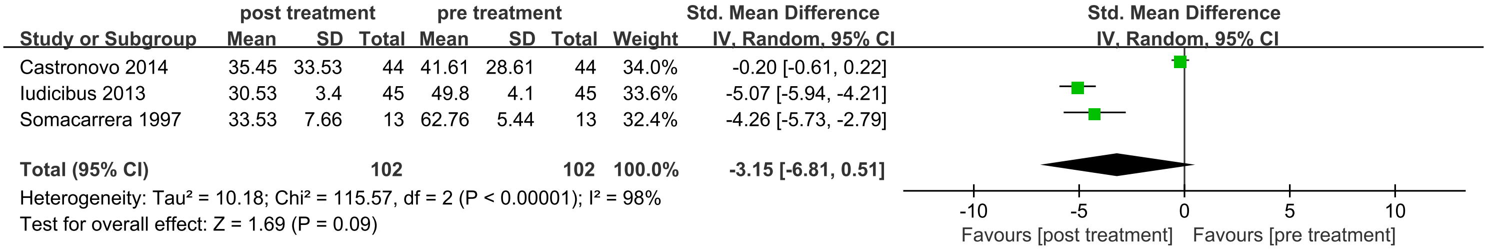

(f)

Study or Subgroup	post treatment Mean	SD	Total	pre treatment Mean	SD	Total	Weight	Std. Mean Difference IV, Random, 95% CI
Aimetti 2008	0.33	0.37	21	1.9	0.56	21	13.3%	-3.25 [-4.19, -2.30]
Gong 2008	1.25	0.5	16	1.64	0.52	16	15.1%	-0.75 [-1.47, -0.03]
Gong 2014	0.69	0.55	15	1.39	0.89	15	14.8%	-0.92 [-1.68, -0.16]
Kantarci 1999	0.69	0.25	15	1.3	0.53	15	14.4%	-1.43 [-2.25, -0.62]
Li 2013	0.48	0.18	12	1.54	0.42	12	10.9%	-3.17 [-4.43, -1.90]
Seymour 1991	0.584	0.26	12	0.835	0.28	12	14.1%	-0.90 [-1.74, -0.05]
Shi 2008	1.91	0.77	57	2.97	1.31	57	17.4%	-0.98 [-1.37, -0.59]
**Total (95% CI)**			**148**			**148**	**100.0%**	**-1.53 [-2.18, -0.88]**

Heterogeneity: $Tau^2 = 0.59$; $Chi^2 = 31.19$, df = 6 ($P < 0.0001$); $I^2 = 81\%$

Test for overall effect: Z = 4.62 ($P < 0.00001$)

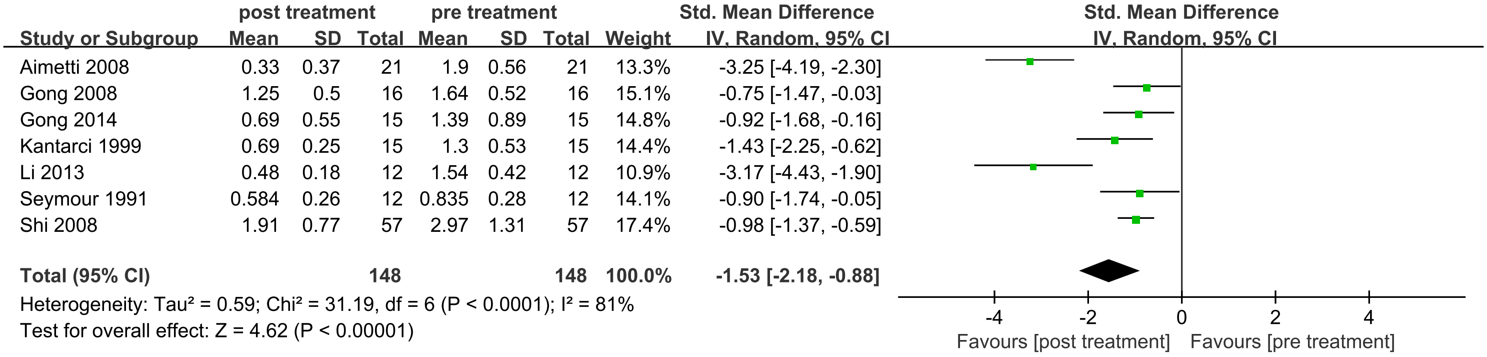

(a)

Study or Subgroup	treatment group Mean	SD	Total	control group Mean	SD	Total	Weight	Std. Mean Difference IV, Random, 95% CI
**1.1.1 Europe**								
Seymour 1991	0.584	0.26	12	1.33	0.56	15	31.6%	-1.60 [-2.48, -0.71]
**Subtotal (95% CI)**			**12**			**15**	**31.6%**	**-1.60 [-2.48, -0.71]**
Heterogeneity: Not applicable								
Test for overall effect: Z = 3.52 (P = 0.0004)								
**1.1.2 Asia**								
Gong 2008	1.25	0.5	16	1.33	0.55	18	35.4%	-0.15 [-0.82, 0.53]
Kantarci 1999	0.69	0.25	15	1.15	0.33	16	33.0%	-1.52 [-2.34, -0.71]
**Subtotal (95% CI)**			**31**			**34**	**68.4%**	**-0.82 [-2.16, 0.53]**
Heterogeneity: Tau² = 0.80; Chi² = 6.51, df = 1 (P = 0.01); I² = 85%								
Test for overall effect: Z = 1.19 (P = 0.23)								
**Total (95% CI)**			**43**			**49**	**100.0%**	**-1.06 [-2.04, -0.07]**
Heterogeneity: Tau² = 0.59; Chi² = 9.37, df = 2 (P = 0.009); I² = 79%								
Test for overall effect: Z = 2.11 (P = 0.04)								
Test for subgroup differences: Chi² = 0.90, df = 1 (P = 0.34), I² = 0%								

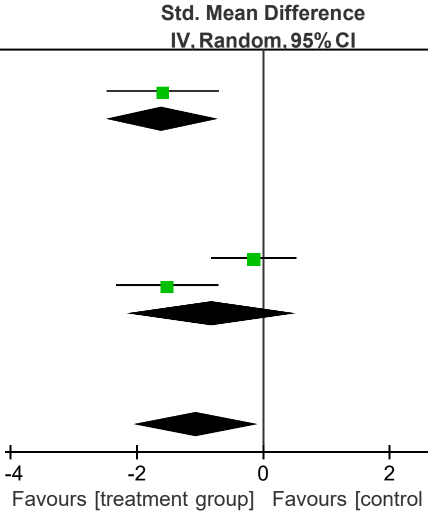

(b)

Study or Subgroup	post treatment Mean	SD	Total	pre treatment Mean	SD	Total	Weight	Std. Mean Difference IV, Random, 95% CI
**3.1.1 Europe**								
Castronovo 2014	14.43	18.78	44	24.3	24.91	44	17.3%	-0.44 [-0.87, -0.02]
Iudicibus 2013	16.57	1.8	45	30.79	3.4	45	16.4%	-5.18 [-6.06, -4.31]
Seymour 1991	21.6	14.6	12	10.6	5.9	12	16.5%	0.95 [0.10, 1.81]
**Subtotal (95% CI)**			**101**			**101**	**50.2%**	**-1.55 [-4.62, 1.52]**
Heterogeneity: Tau² = 7.20; Chi² = 114.00, df = 2 (P < 0.00001); I² = 98%								
Test for overall effect: Z = 0.99 (P = 0.32)								
**3.1.2 Asia**								
Gong 2008	30.7	11.1	16	41.4	8.6	16	16.7%	-1.05 [-1.80, -0.31]
Gong 2014	19.9	9.81	15	42.54	17.18	15	16.5%	-1.57 [-2.41, -0.74]
Kantarci 1999	32.13	11.99	15	53.4	12.26	15	16.5%	-1.71 [-2.56, -0.85]
**Subtotal (95% CI)**			**46**			**46**	**49.8%**	**-1.41 [-1.88, -0.94]**
Heterogeneity: Tau² = 0.00; Chi² = 1.51, df = 2 (P = 0.47); I² = 0%								
Test for overall effect: Z = 5.93 (P < 0.00001)								
**3.1.3 Africa**								
Malek 2019	0	0	1	30.5	0	1		Not estimable
**Subtotal (95% CI)**			**1**			**1**		**Not estimable**
Heterogeneity: Not applicable								
Test for overall effect: Not applicable								
**Total (95% CI)**			**148**			**148**	**100.0%**	**-1.49 [-2.92, -0.06]**
Heterogeneity: Tau² = 3.06; Chi² = 117.85, df = 5 (P < 0.00001); I² = 96%								
Test for overall effect: Z = 2.04 (P = 0.04)								
Test for subgroup differences: Chi² = 0.01, df = 1 (P = 0.93), I² = 0%								

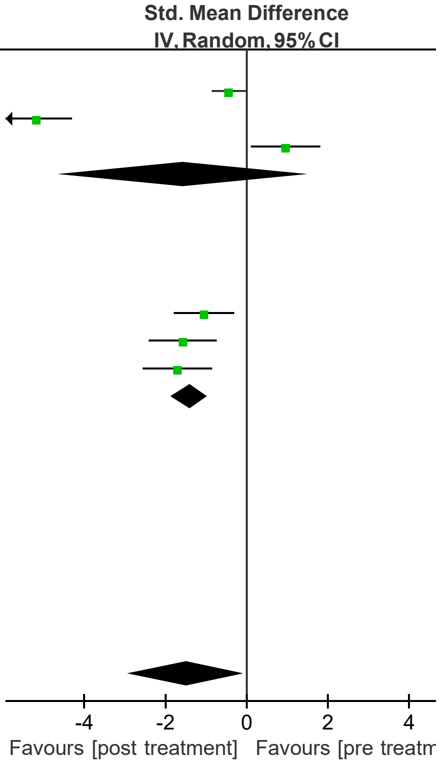

(c)

Study or Subgroup	post treatment Mean	SD	Total	pre treatment Mean	SD	Total	Weight	Std. Mean Difference IV, Random, 95% CI
**4.1.1 Europe**								
Aimetti 2005	2.1	0.86	21	3.9	1.4	21	18.1%	-1.52 [-2.21, -0.83]
Romano 2015	4.2	1.5	1	8.1	2.9	1		Not estimable
**Subtotal (95% CI)**			**22**			**22**	**18.1%**	**-1.52 [-2.21, -0.83]**
Heterogeneity: Not applicable								
Test for overall effect: Z = 4.29 (P < 0.0001)								
**4.1.2 Asia**								
Gong 2008	2.62	0.96	16	3.44	0.85	16	17.9%	-0.88 [-1.61, -0.15]
Gong 2014	2.2	0.47	15	3.38	0.79	15	17.2%	-1.77 [-2.63, -0.90]
Kantarci 1999	2.97	0.8	15	3.75	1.2	15	17.8%	-0.74 [-1.49, -0.00]
Li 2013	2.25	0.27	12	5.03	0.51	12	9.6%	-6.58 [-8.77, -4.39]
Shi 2008	2.91	1.14	57	3.03	1.08	57	19.5%	-0.11 [-0.47, 0.26]
**Subtotal (95% CI)**			**115**			**115**	**81.9%**	**-1.56 [-2.68, -0.45]**
Heterogeneity: Tau² = 1.34; Chi² = 43.36, df = 4 (P < 0.00001); I² = 91%								
Test for overall effect: Z = 2.76 (P = 0.006)								
**Total (95% CI)**			**137**			**137**	**100.0%**	**-1.52 [-2.46, -0.58]**
Heterogeneity: Tau² = 1.14; Chi² = 49.19, df = 5 (P < 0.00001); I² = 90%								
Test for overall effect: Z = 3.18 (P = 0.001)								
Test for subgroup differences: Chi² = 0.00, df = 1 (P = 0.95), I² = 0%								

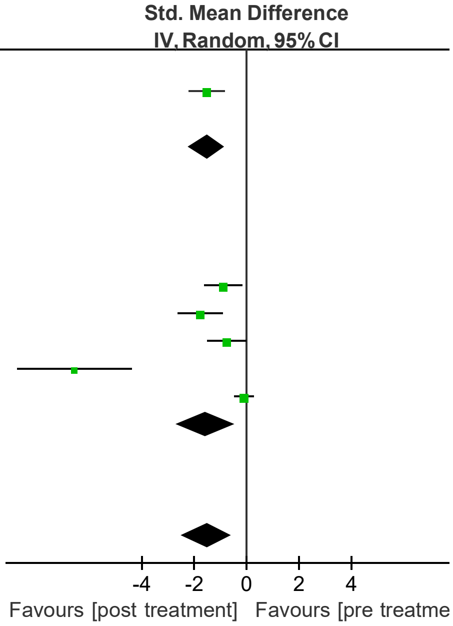

(d)

Study or Subgroup	post treatment Mean	post treatment SD	post treatment Total	pre treatment Mean	pre treatment SD	pre treatment Total	Weight	Std. Mean Difference IV, Random, 95% CI
**7.1.1 Europe**								
Aimetti 2008	0.33	0.37	21	1.9	0.56	21	13.3%	-3.25 [-4.19, -2.30]
Seymour 1991	0.584	0.26	12	0.835	0.28	12	14.1%	-0.90 [-1.74, -0.05]
**Subtotal (95% CI)**			**33**			**33**	**27.4%**	**-2.06 [-4.36, 0.24]**
Heterogeneity: Tau² = 2.55; Chi² = 13.12, df = 1 (P = 0.0003); I² = 92%								
Test for overall effect: Z = 1.76 (P = 0.08)								
**7.1.2 Asia**								
Gong 2008	1.25	0.5	16	1.64	0.52	16	15.1%	-0.75 [-1.47, -0.03]
Gong 2014	0.69	0.55	15	1.39	0.89	15	14.8%	-0.92 [-1.68, -0.16]
Kantarci 1999	0.69	0.25	15	1.3	0.53	15	14.4%	-1.43 [-2.25, -0.62]
Li 2013	0.48	0.18	12	1.54	0.42	12	10.9%	-3.17 [-4.43, -1.90]
Shi 2008	1.91	0.77	57	2.97	1.31	57	17.4%	-0.98 [-1.37, -0.59]
**Subtotal (95% CI)**			**115**			**115**	**72.6%**	**-1.28 [-1.85, -0.71]**
Heterogeneity: Tau² = 0.27; Chi² = 12.41, df = 4 (P = 0.01); I² = 68%								
Test for overall effect: Z = 4.38 (P < 0.0001)								
**Total (95% CI)**			**148**			**148**	**100.0%**	**-1.53 [-2.18, -0.88]**
Heterogeneity: Tau² = 0.59; Chi² = 31.19, df = 6 (P < 0.0001); I² = 81%								
Test for overall effect: Z = 4.62 (P < 0.00001)								
Test for subgroup differences: Chi² = 0.42, df = 1 (P = 0.52), I² = 0%								

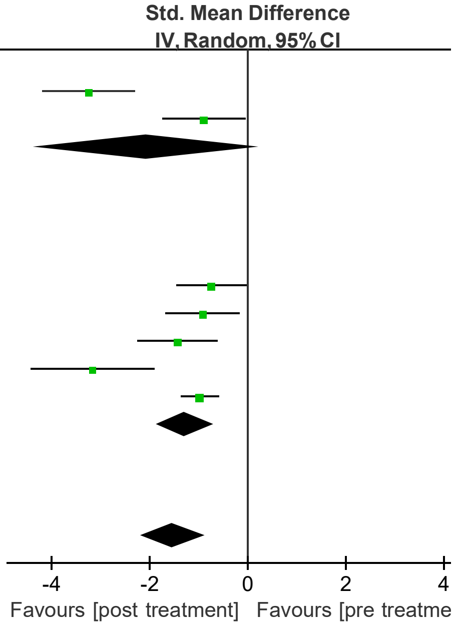

(a)

Study or Subgroup	post treatment Mean	SD	Total	pre treatment Mean	SD	Total	Weight	Std. Mean Difference IV, Random, 95% CI
**3.3.1 M/F > 1**								
Castronovo 2014	14.43	18.78	44	24.3	24.91	44	17.3%	-0.44 [-0.87, -0.02]
Gong 2008	30.7	11.1	16	41.4	8.6	16	16.7%	-1.05 [-1.80, -0.31]
Iudicibus 2013	16.57	1.8	45	30.79	3.4	45	16.4%	-5.18 [-6.06, -4.31]
Kantarci 1999	32.13	11.99	15	53.4	12.26	15	16.5%	-1.71 [-2.56, -0.85]
Seymour 1991	21.6	14.6	12	10.6	5.9	12	16.5%	0.95 [0.10, 1.81]
**Subtotal (95% CI)**			**132**			**132**	**83.5%**	**-1.47 [-3.19, 0.24]**
Heterogeneity: Tau² = 3.69; Chi² = 116.55, df = 4 (P < 0.00001); I² = 97%								
Test for overall effect: Z = 1.68 (P = 0.09)								
**3.3.2 M/F < 1**								
Gong 2014	19.9	9.81	15	42.54	17.18	15	16.5%	-1.57 [-2.41, -0.74]
Malek 2019	0	0	1	30.5	0	1		Not estimable
**Subtotal (95% CI)**			**16**			**16**	**16.5%**	**-1.57 [-2.41, -0.74]**
Heterogeneity: Not applicable								
Test for overall effect: Z = 3.70 (P = 0.0002)								
**Total (95% CI)**			**148**			**148**	**100.0%**	**-1.49 [-2.92, -0.06]**
Heterogeneity: Tau² = 3.06; Chi² = 117.85, df = 5 (P < 0.00001); I² = 96%								
Test for overall effect: Z = 2.04 (P = 0.04)								
Test for subgroup differences: Chi² = 0.01, df = 1 (P = 0.92), I² = 0%								

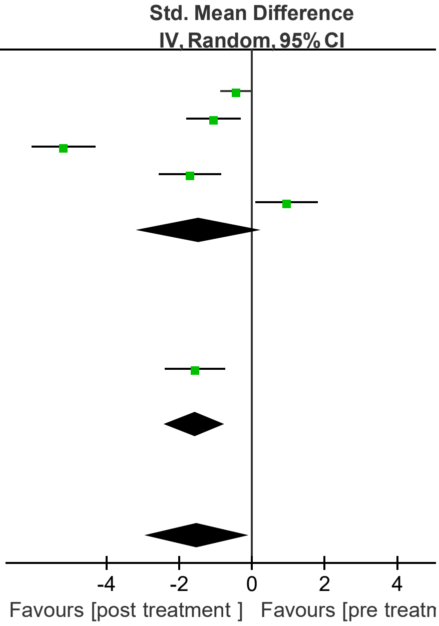

(b)

Study or Subgroup	post treatment Mean	SD	Total	pre treatment Mean	SD	Total	Weight	Std. Mean Difference IV, Random, 95% CI
**4.2.1 M/F > 1**								
Aimetti 2005	2.1	0.86	21	3.9	1.4	21	18.1%	-1.52 [-2.21, -0.83]
Gong 2008	2.62	0.96	16	3.44	0.85	16	17.9%	-0.88 [-1.61, -0.15]
Kantarci 1999	2.97	0.8	15	3.75	1.2	15	17.8%	-0.74 [-1.49, -0.00]
Li 2013	2.25	0.27	12	5.03	0.51	12	9.6%	-6.58 [-8.77, -4.39]
Romano 2015	4.2	1.5	1	8.1	2.9	1		Not estimable
Shi 2008	2.91	1.14	57	3.03	1.08	57	19.5%	-0.11 [-0.47, 0.26]
**Subtotal (95% CI)**			**122**			**122**	**82.8%**	**-1.48 [-2.54, -0.43]**
Heterogeneity: Tau² = 1.20; Chi² = 43.04, df = 4 (P < 0.00001); I² = 91%								
Test for overall effect: Z = 2.76 (P = 0.006)								
**4.2.2 M/F < 1**								
Gong 2014	2.2	0.47	15	3.38	0.79	15	17.2%	-1.77 [-2.63, -0.90]
**Subtotal (95% CI)**			**15**			**15**	**17.2%**	**-1.77 [-2.63, -0.90]**
Heterogeneity: Not applicable								
Test for overall effect: Z = 4.02 (P < 0.0001)								
**Total (95% CI)**			**137**			**137**	**100.0%**	**-1.52 [-2.46, -0.58]**
Heterogeneity: Tau² = 1.14; Chi² = 49.19, df = 5 (P < 0.00001); I² = 90%								
Test for overall effect: Z = 3.18 (P = 0.001)								
Test for subgroup differences: Chi² = 0.17, df = 1 (P = 0.68), I² = 0%								

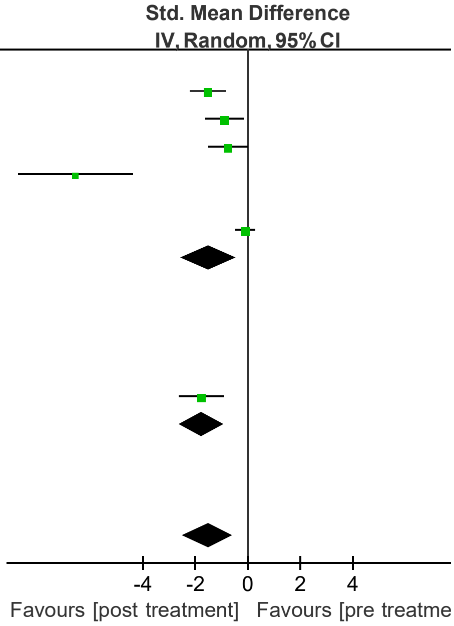

(c)

Study or Subgroup	post treatment Mean	post treatment SD	post treatment Total	pre treatment Mean	pre treatment SD	pre treatment Total	Weight	Std. Mean Difference IV, Random, 95% CI
**7.2.1 M/F > 1**								
Aimetti 2008	0.33	0.37	21	1.9	0.56	21	13.3%	-3.25 [-4.19, -2.30]
Gong 2008	1.25	0.5	16	1.64	0.52	16	15.1%	-0.75 [-1.47, -0.03]
Kantarci 1999	0.69	0.25	15	1.3	0.53	15	14.4%	-1.43 [-2.25, -0.62]
Li 2013	0.48	0.18	12	1.54	0.42	12	10.9%	-3.17 [-4.43, -1.90]
Seymour 1991	0.584	0.26	12	0.835	0.28	12	14.1%	-0.90 [-1.74, -0.05]
Shi 2008	1.91	0.77	57	2.97	1.31	57	17.4%	-0.98 [-1.37, -0.59]
**Subtotal (95% CI)**			**133**			**133**	**85.2%**	**-1.65 [-2.41, -0.89]**
Heterogeneity: Tau² = 0.72; Chi² = 30.41, df = 5 (P < 0.0001); I² = 84%								
Test for overall effect: Z = 4.24 (P < 0.0001)								
**7.2.2 M/F < 1**								
Gong 2014	0.69	0.55	15	1.39	0.89	15	14.8%	-0.92 [-1.68, -0.16]
**Subtotal (95% CI)**			**15**			**15**	**14.8%**	**-0.92 [-1.68, -0.16]**
Heterogeneity: Not applicable								
Test for overall effect: Z = 2.38 (P = 0.02)								
**Total (95% CI)**			**148**			**148**	**100.0%**	**-1.53 [-2.18, -0.88]**
Heterogeneity: Tau² = 0.59; Chi² = 31.19, df = 6 (P < 0.0001); I² = 81%								
Test for overall effect: Z = 4.62 (P < 0.00001)								
Test for subgroup differences: Chi² = 1.76, df = 1 (P = 0.18), I² = 43.3%								

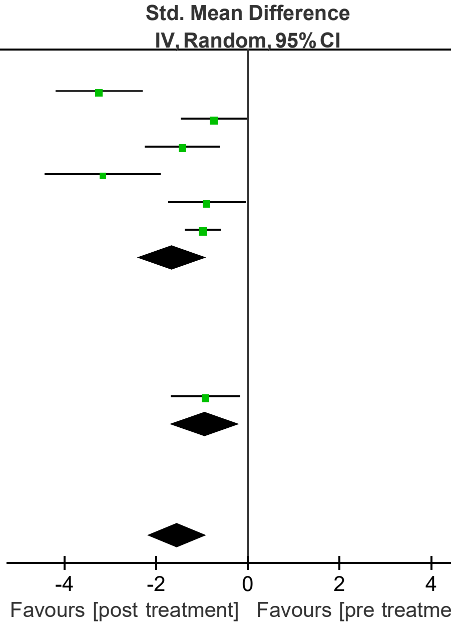

(a)

Study or Subgroup	treatment group Mean	SD	Total	control group Mean	SD	Total	Weight	Std. Mean Difference IV, Random, 95% CI
**1.2.1 yes**								
Gong 2008	1.25	0.5	16	1.33	0.55	18	35.4%	-0.15 [-0.82, 0.53]
**Subtotal (95% CI)**			**16**			**18**	**35.4%**	**-0.15 [-0.82, 0.53]**
Heterogeneity: Not applicable								
Test for overall effect: Z = 0.43 (P = 0.67)								
**1.2.2 no**								
Kantarci 1999	0.69	0.25	15	1.15	0.33	16	33.0%	-1.52 [-2.34, -0.71]
Seymour 1991	0.584	0.26	12	1.33	0.56	15	31.6%	-1.60 [-2.48, -0.71]
**Subtotal (95% CI)**			**27**			**31**	**64.6%**	**-1.56 [-2.16, -0.96]**
Heterogeneity: Tau² = 0.00; Chi² = 0.01, df = 1 (P = 0.91); I² = 0%								
Test for overall effect: Z = 5.09 (P < 0.00001)								
**Total (95% CI)**			**43**			**49**	**100.0%**	**-1.06 [-2.04, -0.07]**
Heterogeneity: Tau² = 0.59; Chi² = 9.37, df = 2 (P = 0.009); I² = 79%								
Test for overall effect: Z = 2.11 (P = 0.04)								
Test for subgroup differences: Chi² = 9.36, df = 1 (P = 0.002), I² = 89.3%								

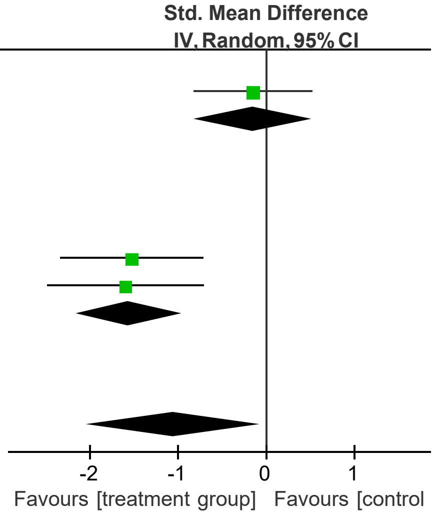

(b)

Study or Subgroup	post treatment Mean	post treatment SD	post treatment Total	pre treatment Mean	pre treatment SD	pre treatment Total	Weight	Std. Mean Difference IV, Random, 95% CI
**2.1.1 yes**								
Aimetti 2005	0.56	0.83	21	2.38	1.92	21	50.9%	-1.21 [-1.87, -0.54]
**Subtotal (95% CI)**			**21**			**21**	**50.9%**	**-1.21 [-1.87, -0.54]**
Heterogeneity: Not applicable								
Test for overall effect: Z = 3.57 (P = 0.0004)								
**2.1.2 no**								
Aimetti 2008	0.48	0.68	21	4.24	0.24	21	49.1%	-7.23 [-8.97, -5.50]
Romano 2015	0.3	0.5	1	3.2	1.5	1		Not estimable
**Subtotal (95% CI)**			**22**			**22**	**49.1%**	**-7.23 [-8.97, -5.50]**
Heterogeneity: Not applicable								
Test for overall effect: Z = 8.18 (P < 0.00001)								
**Total (95% CI)**			**43**			**43**	**100.0%**	**-4.17 [-10.07, 1.74]**

Heterogeneity: Tau² = 17.72; Chi² = 40.49, df = 1 (P < 0.00001); I² = 98%
Test for overall effect: Z = 1.38 (P = 0.17)
Test for subgroup differences: Chi² = 40.49, df = 1 (P < 0.00001), I² = 97.5%

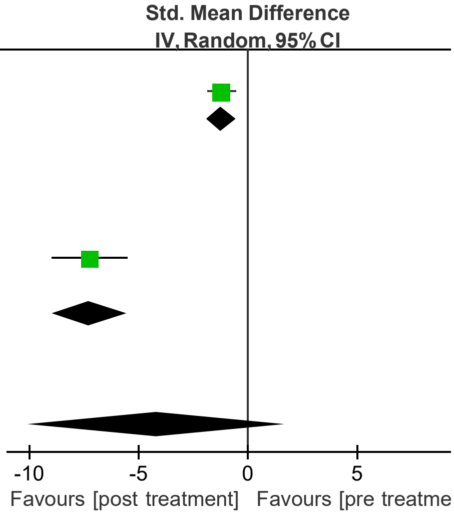

(c)

Study or Subgroup	post treatment Mean	post treatment SD	post treatment Total	pre treatment Mean	pre treatment SD	pre treatment Total	Weight	Std. Mean Difference IV, Random, 95% CI
**3.2.1 yes**								
Castronovo 2014	14.43	18.78	44	24.3	24.91	44	17.3%	-0.44 [-0.87, -0.02]
Gong 2008	30.7	11.1	16	41.4	8.6	16	16.7%	-1.05 [-1.80, -0.31]
Gong 2014	19.9	9.81	15	42.54	17.18	15	16.5%	-1.57 [-2.41, -0.74]
Iudicibus 2013	16.57	1.8	45	30.79	3.4	45	16.4%	-5.18 [-6.06, -4.31]
Malek 2019	0	0	1	30.5	0	1		Not estimable
**Subtotal (95% CI)**			**121**			**121**	**67.0%**	**-2.04 [-3.95, -0.14]**
Heterogeneity: Tau² = 3.64; Chi² = 91.75, df = 3 (P < 0.00001); I² = 97%								
Test for overall effect: Z = 2.10 (P = 0.04)								
**3.2.2 no**								
Kantarci 1999	32.13	11.99	15	53.4	12.26	15	16.5%	-1.71 [-2.56, -0.85]
Seymour 1991	21.6	14.6	12	10.6	5.9	12	16.5%	0.95 [0.10, 1.81]
**Subtotal (95% CI)**			**27**			**27**	**33.0%**	**-0.38 [-2.98, 2.23]**
Heterogeneity: Tau² = 3.35; Chi² = 18.70, df = 1 (P < 0.0001); I² = 95%								
Test for overall effect: Z = 0.28 (P = 0.78)								
**Total (95% CI)**			**148**			**148**	**100.0%**	**-1.49 [-2.92, -0.06]**
Heterogeneity: Tau² = 3.06; Chi² = 117.85, df = 5 (P < 0.00001); I² = 96%								
Test for overall effect: Z = 2.04 (P = 0.04)								
Test for subgroup differences: Chi² = 1.02, df = 1 (P = 0.31), I² = 2.1%								

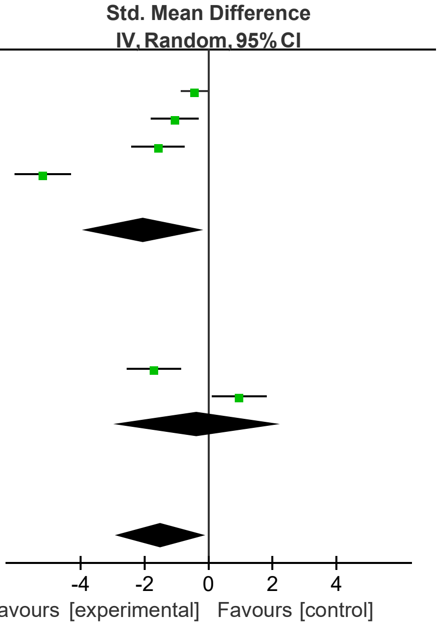

(d)

Study or Subgroup	post treatment Mean	SD	Total	pre treatment Mean	SD	Total	Weight	Std. Mean Difference IV, Random, 95% CI
**4.3.1 yes**								
Aimetti 2005	2.1	0.86	21	3.9	1.4	21	18.1%	-1.52 [-2.21, -0.83]
Gong 2008	2.62	0.96	16	3.44	0.85	16	17.9%	-0.88 [-1.61, -0.15]
Gong 2014	2.2	0.47	15	3.38	0.79	15	17.2%	-1.77 [-2.63, -0.90]
Shi 2008	2.91	1.14	57	3.03	1.08	57	19.5%	-0.11 [-0.47, 0.26]
**Subtotal (95% CI)**			**109**			**109**	**72.6%**	**-1.02 [-1.87, -0.18]**
Heterogeneity: Tau² = 0.62; Chi² = 21.22, df = 3 (P < 0.0001); I² = 86%								
Test for overall effect: Z = 2.38 (P = 0.02)								
**4.3.2 no**								
Kantarci 1999	2.97	0.8	15	3.75	1.2	15	17.8%	-0.74 [-1.49, -0.00]
Li 2013	2.25	0.27	12	5.03	0.51	12	9.6%	-6.58 [-8.77, -4.39]
Romano 2015	4.2	1.5	1	8.1	2.9	1		Not estimable
**Subtotal (95% CI)**			**28**			**28**	**27.4%**	**-3.57 [-9.28, 2.15]**
Heterogeneity: Tau² = 16.32; Chi² = 24.50, df = 1 (P < 0.00001); I² = 96%								
Test for overall effect: Z = 1.22 (P = 0.22)								
**Total (95% CI)**			**137**			**137**	**100.0%**	**-1.52 [-2.46, -0.58]**
Heterogeneity: Tau² = 1.14; Chi² = 49.19, df = 5 (P < 0.00001); I² = 90%								
Test for overall effect: Z = 3.18 (P = 0.001)								
Test for subgroup differences: Chi² = 0.75, df = 1 (P = 0.39), I² = 0%								

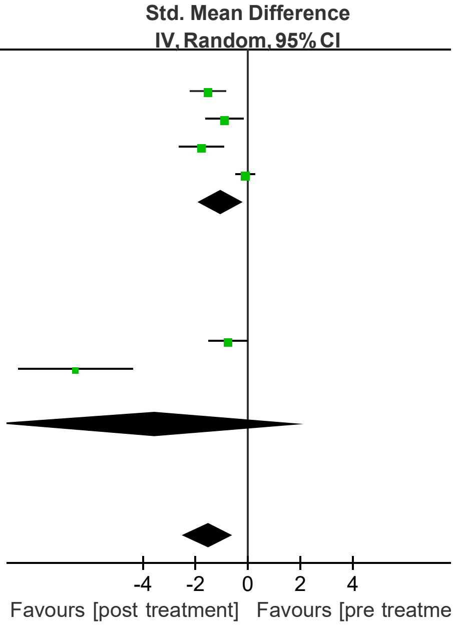

(e)

Study or Subgroup	post treatment Mean	SD	Total	pre treatment Mean	SD	Total	Weight	Std. Mean Difference IV, Random, 95% CI
**5.1.1 yes**								
Castronovo 2014	13.37	14.38	44	23.38	27.14	44	33.8%	-0.46 [-0.88, -0.03]
Iudicibus 2013	17.41	2.5	45	33.42	4.3	45	33.1%	-4.51 [-5.30, -3.72]
**Subtotal (95% CI)**			**89**			**89**	**67.0%**	**-2.47 [-6.45, 1.50]**
Heterogeneity: Tau² = 8.12; Chi² = 78.54, df = 1 (P < 0.00001); I² = 99%								
Test for overall effect: Z = 1.22 (P = 0.22)								
**5.1.2 no**								
Seymour 1991	11.6	16.4	12	0.25	12.56	12	33.0%	0.75 [-0.08, 1.58]
**Subtotal (95% CI)**			**12**			**12**	**33.0%**	**0.75 [-0.08, 1.58]**
Heterogeneity: Not applicable								
Test for overall effect: Z = 1.76 (P = 0.08)								
**Total (95% CI)**			**101**			**101**	**100.0%**	**-1.40 [-4.12, 1.32]**
Heterogeneity: Tau² = 5.66; Chi² = 99.09, df = 2 (P < 0.00001); I² = 98%								
Test for overall effect: Z = 1.01 (P = 0.31)								
Test for subgroup differences: Chi² = 2.42, df = 1 (P = 0.12), I² = 58.6%								

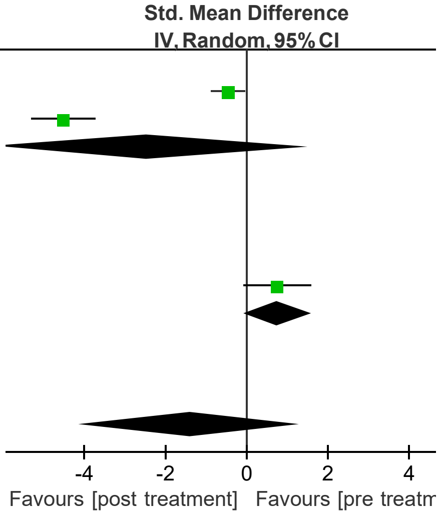

(f)

Study or Subgroup	post treatment Mean	SD	Total	pre treatment Mean	SD	Total	Weight	Std. Mean Difference IV, Random, 95% CI
**6.1.1 yes**								
Castronovo 2014	35.45	33.53	44	41.61	28.61	44	34.0%	-0.20 [-0.61, 0.22]
Iudicibus 2013	30.53	3.4	45	49.8	4.1	45	33.6%	-5.07 [-5.94, -4.21]
**Subtotal (95% CI)**			**89**			**89**	**67.6%**	**-2.62 [-7.40, 2.16]**
Heterogeneity: Tau² = 11.77; Chi² = 99.25, df = 1 (P < 0.00001); I² = 99%								
Test for overall effect: Z = 1.07 (P = 0.28)								
**6.1.2 no**								
Somacarrera 1997	33.53	7.66	13	62.76	5.44	13	32.4%	-4.26 [-5.73, -2.79]
**Subtotal (95% CI)**			**13**			**13**	**32.4%**	**-4.26 [-5.73, -2.79]**
Heterogeneity: Not applicable								
Test for overall effect: Z = 5.67 (P < 0.00001)								
**Total (95% CI)**			**102**			**102**	**100.0%**	**-3.15 [-6.81, 0.51]**
Heterogeneity: Tau² = 10.18; Chi² = 115.57, df = 2 (P < 0.00001); I² = 98%								
Test for overall effect: Z = 1.69 (P = 0.09)								
Test for subgroup differences: Chi² = 0.41, df = 1 (P = 0.52), I² = 0%								

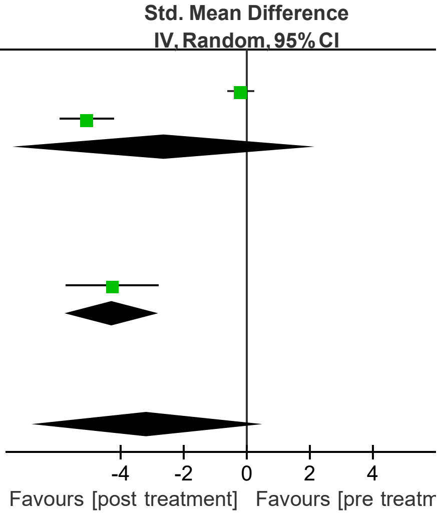

(g)

Study or Subgroup	post treatment Mean	SD	Total	pre treatment Mean	SD	Total	Weight	Std. Mean Difference IV, Random, 95% CI
**7.3.1 yes**								
Gong 2008	1.25	0.5	16	1.64	0.52	16	15.1%	-0.75 [-1.47, -0.03]
Gong 2014	0.69	0.55	15	1.39	0.89	15	14.8%	-0.92 [-1.68, -0.16]
Shi 2008	1.91	0.77	57	2.97	1.31	57	17.4%	-0.98 [-1.37, -0.59]
**Subtotal (95% CI)**			**88**			**88**	**47.3%**	**-0.93 [-1.24, -0.61]**
Heterogeneity: Tau² = 0.00; Chi² = 0.32, df = 2 (P = 0.85); I² = 0%								
Test for overall effect: Z = 5.81 (P < 0.00001)								
**7.3.2 no**								
Aimetti 2008	0.33	0.37	21	1.9	0.56	21	13.3%	-3.25 [-4.19, -2.30]
Kantarci 1999	0.69	0.25	15	1.3	0.53	15	14.4%	-1.43 [-2.25, -0.62]
Li 2013	0.48	0.18	12	1.54	0.42	12	10.9%	-3.17 [-4.43, -1.90]
Seymour 1991	0.584	0.26	12	0.835	0.28	12	14.1%	-0.90 [-1.74, -0.05]
**Subtotal (95% CI)**			**60**			**60**	**52.7%**	**-2.14 [-3.30, -0.97]**
Heterogeneity: Tau² = 1.17; Chi² = 18.23, df = 3 (P = 0.0004); I² = 84%								
Test for overall effect: Z = 3.59 (P = 0.0003)								
**Total (95% CI)**			**148**			**148**	**100.0%**	**-1.53 [-2.18, -0.88]**
Heterogeneity: Tau² = 0.59; Chi² = 31.19, df = 6 (P < 0.0001); I² = 81%								
Test for overall effect: Z = 4.62 (P < 0.00001)								
Test for subgroup differences: Chi² = 3.86, df = 1 (P = 0.05), I² = 74.1%								

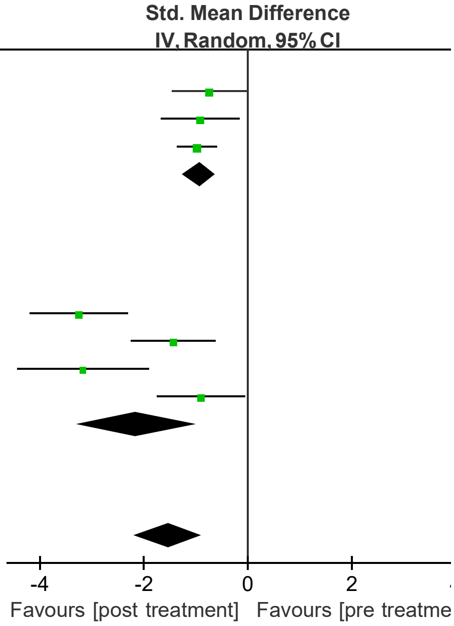

(a)
treatment group vs. control group
outcome: HI %

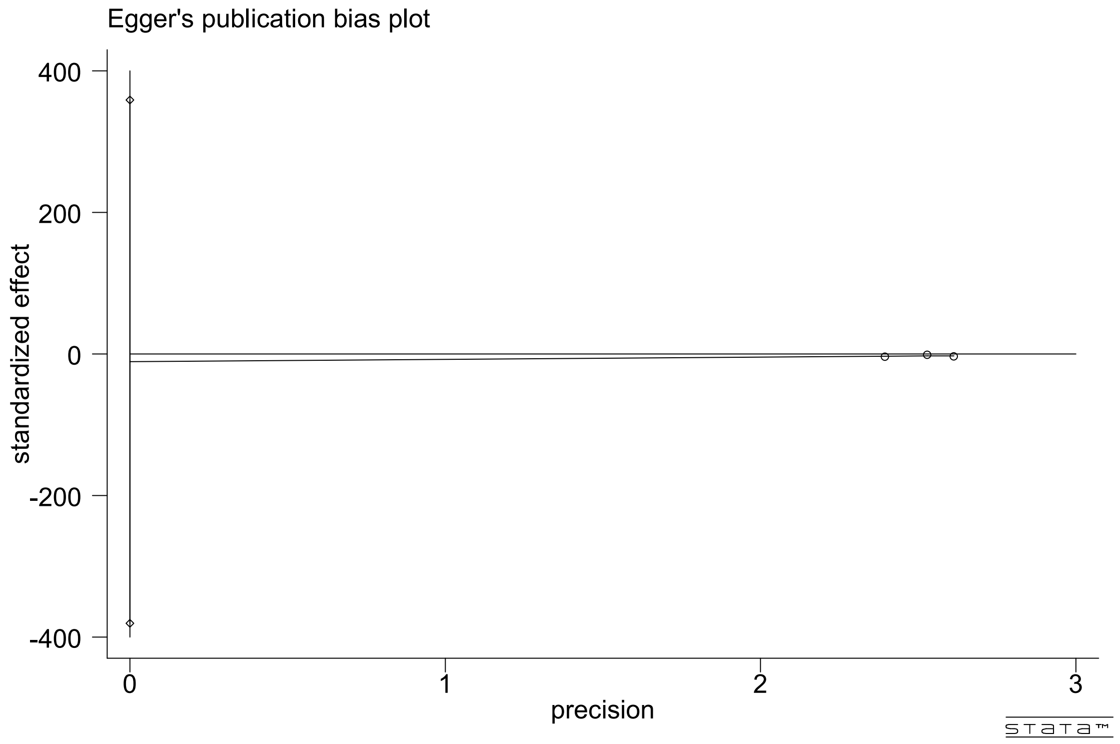

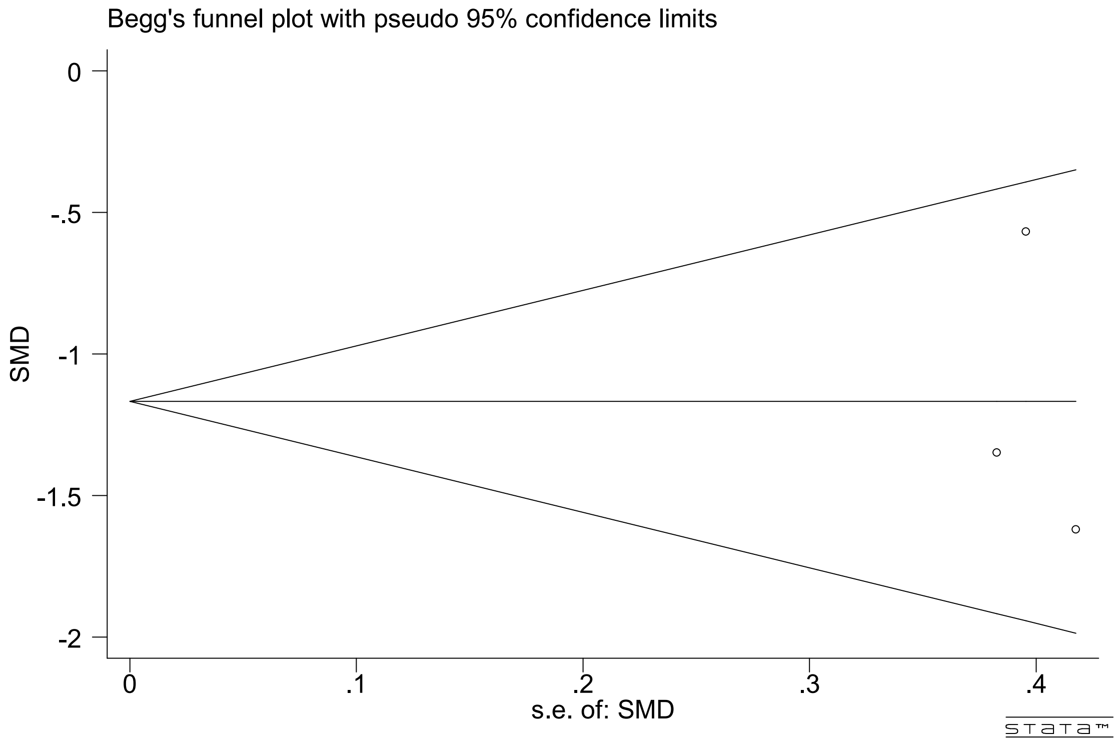

Begg's funnel plot with pseudo 95% confidence limits
0
-.5
-1
-1.5
-2
SMD
0
.1
.2
.3
.4
s.e. of: SMD
stata™

(b)
treatment group vs. control group
outcome: PI index

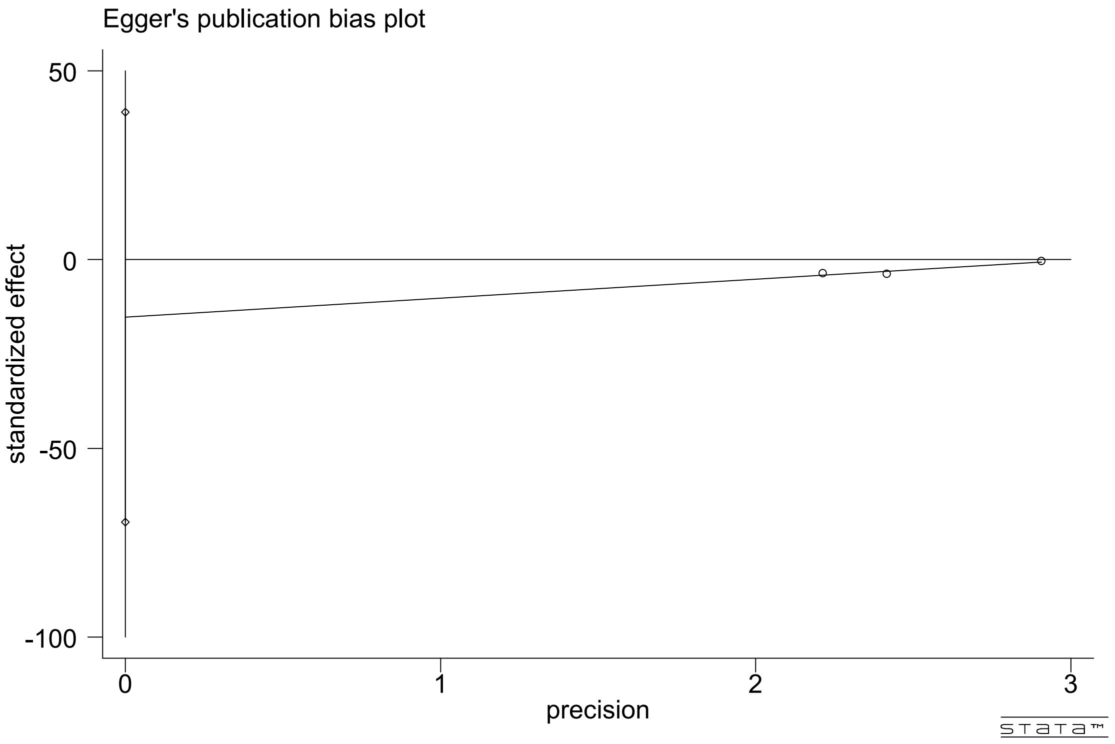

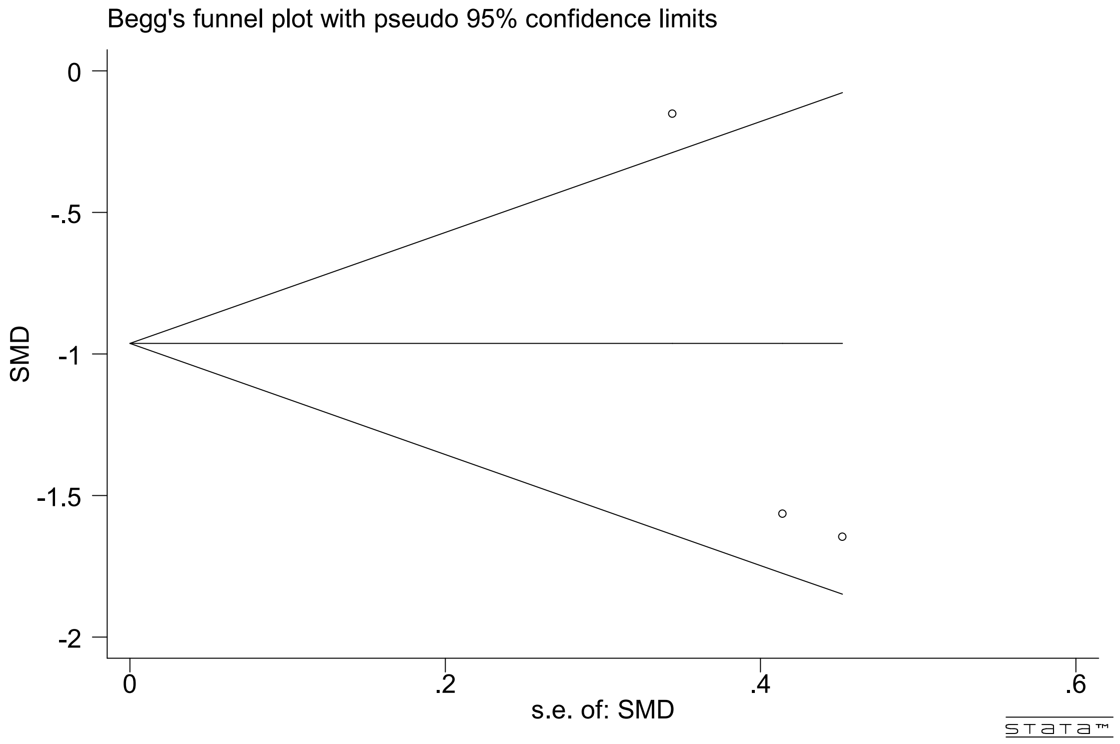
Begg's funnel plot with pseudo 95% confidence limits
0
-.5
-1
-1.5
-2
SMD
0
.2
.4
.6
s.e. of: SMD
stata™

(c)
post-treatment vs. pre-treatment
outcome: GO score

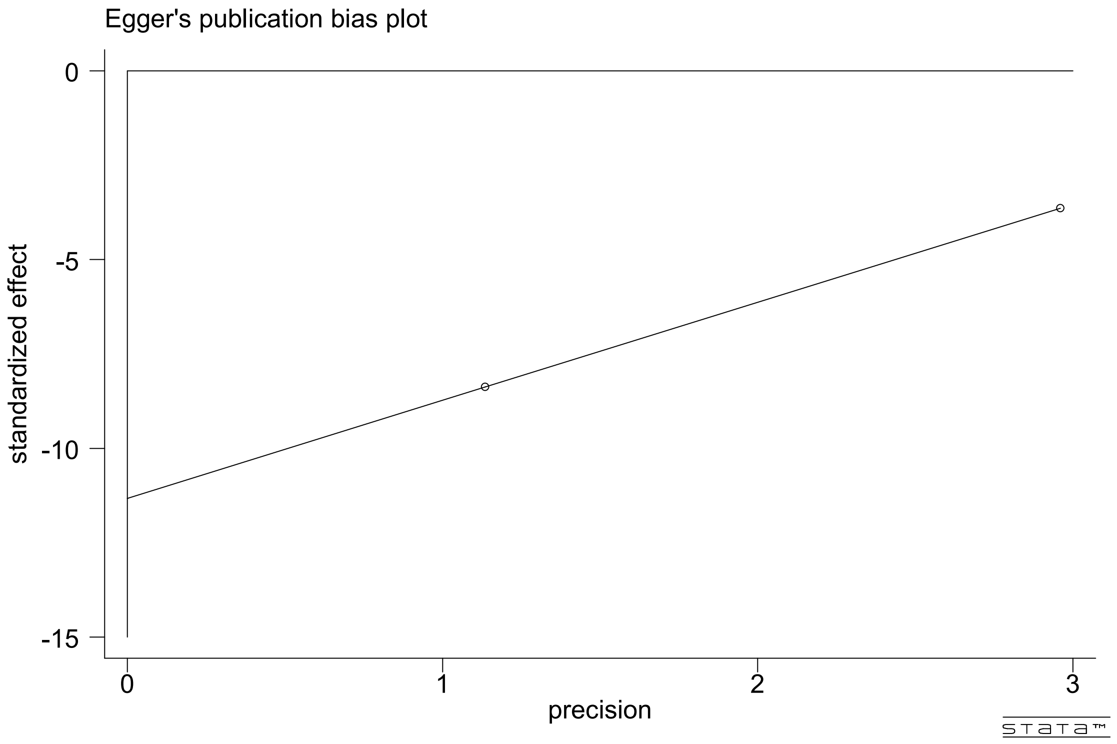

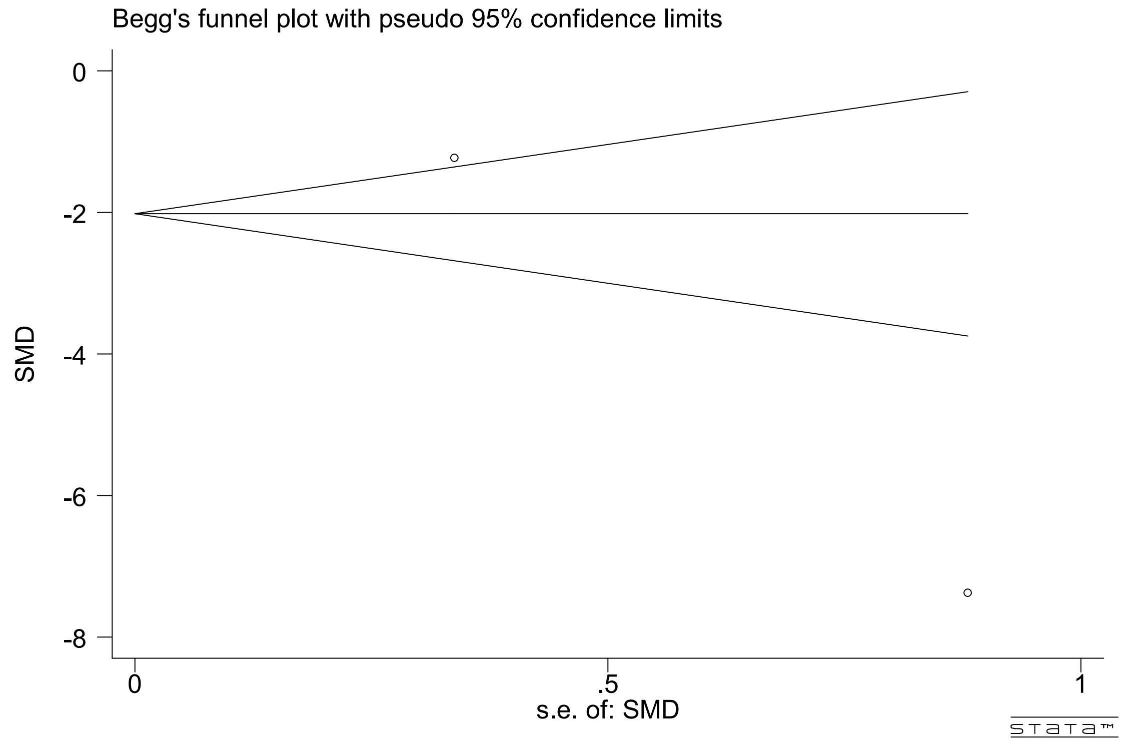
Begg's funnel plot with pseudo 95% confidence limits
SMD
0
-2
-4
-6
-8
0
.5
1
s.e. of: SMD
stata™

(d)
post-treatment vs. pre-treatment
outcome: HI %

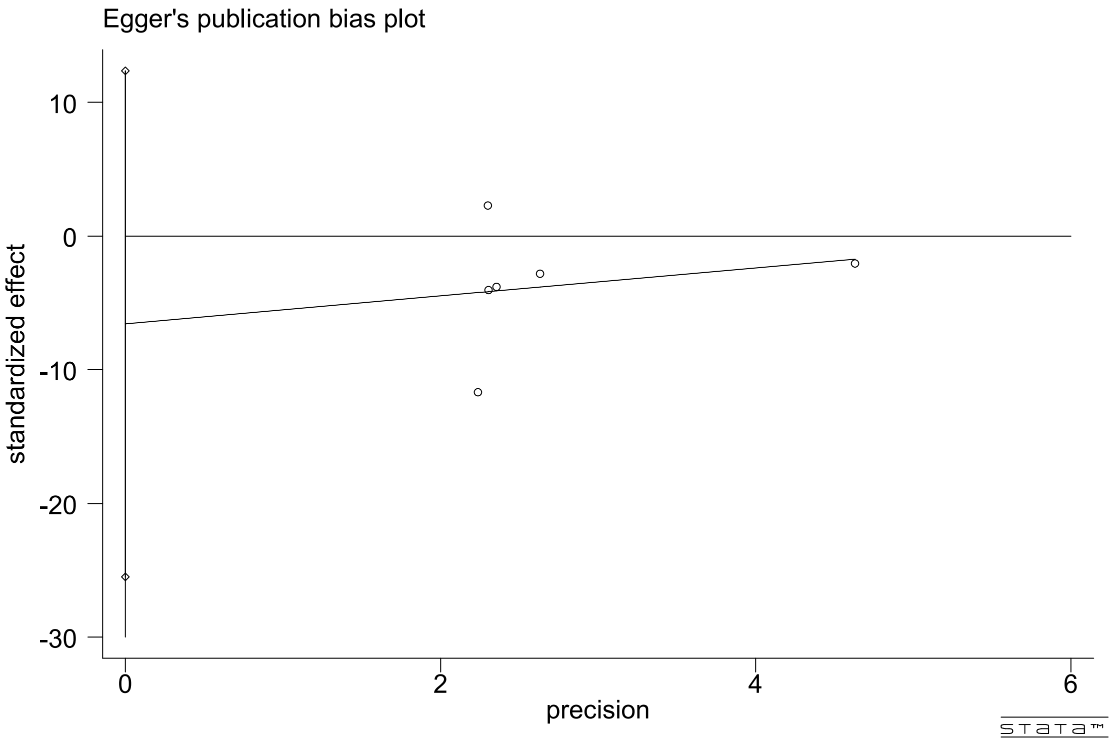

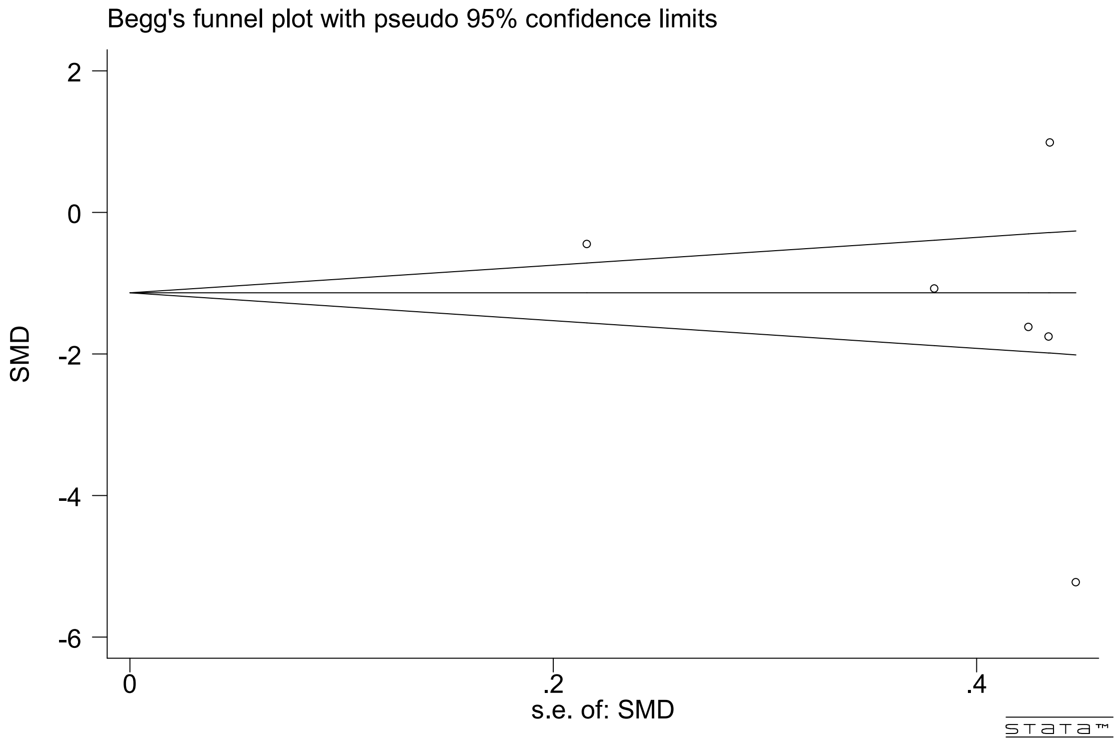

Begg's funnel plot with pseudo 95% confidence limits
2
0
-2
-4
-6
SMD
0
.2
.4
s.e. of: SMD
stata™

(e)
post-treatment vs. pre-treatment
outcome: PD mm

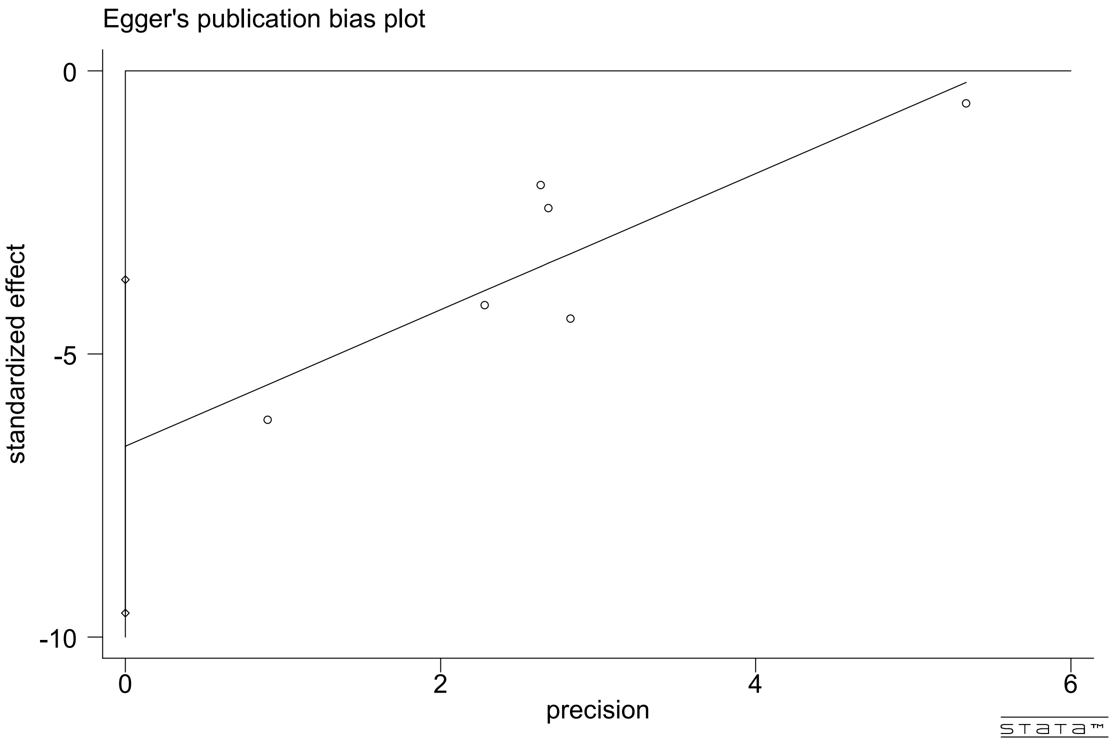

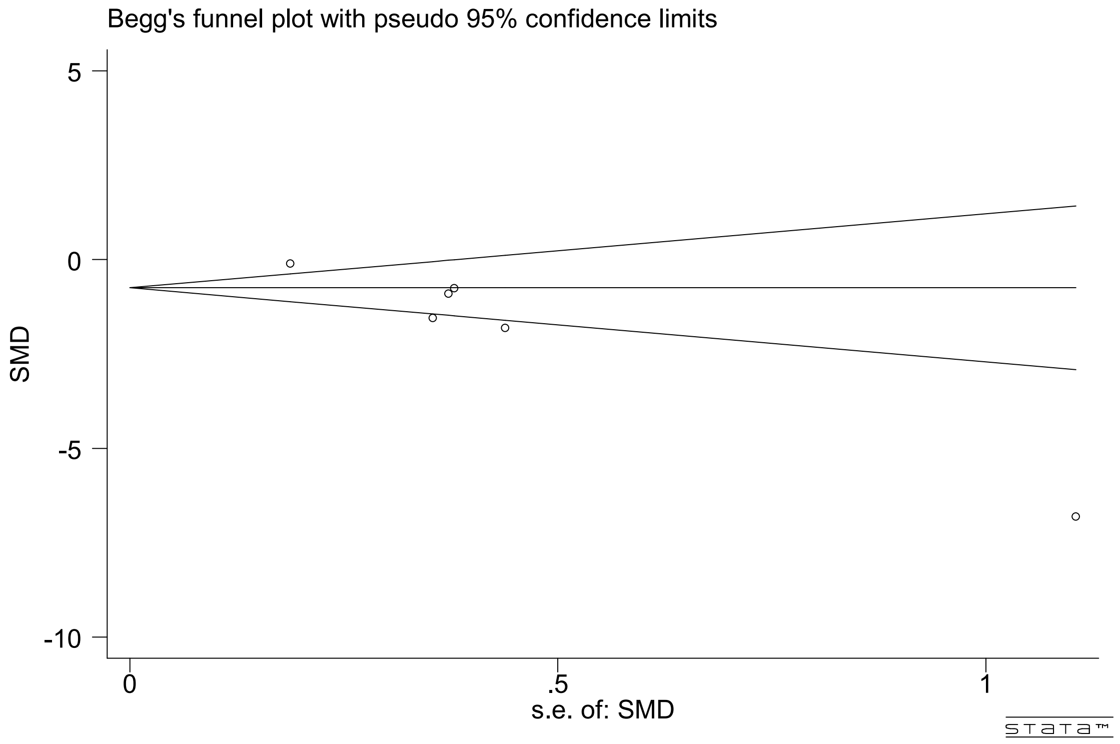
Begg's funnel plot with pseudo 95% confidence limits
5
0
-5
-10
SMD
0
.5
1
s.e. of: SMD
stata™

(f)
post-treatment vs. pre-treatment
outcome: PD %

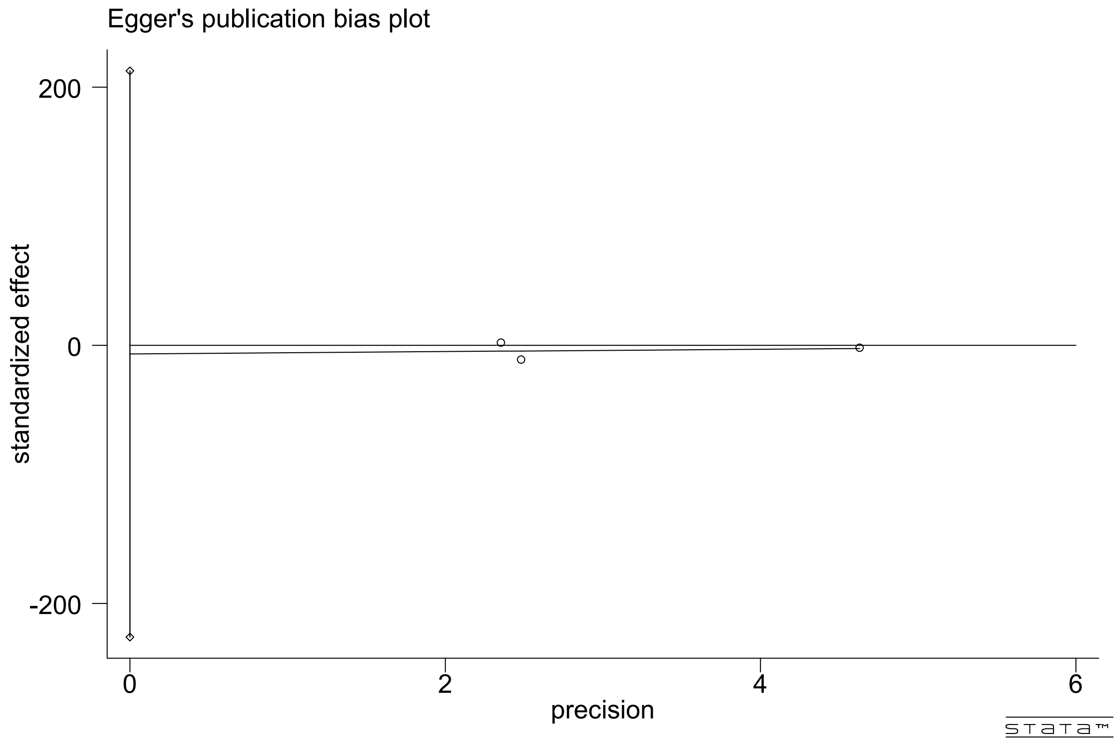

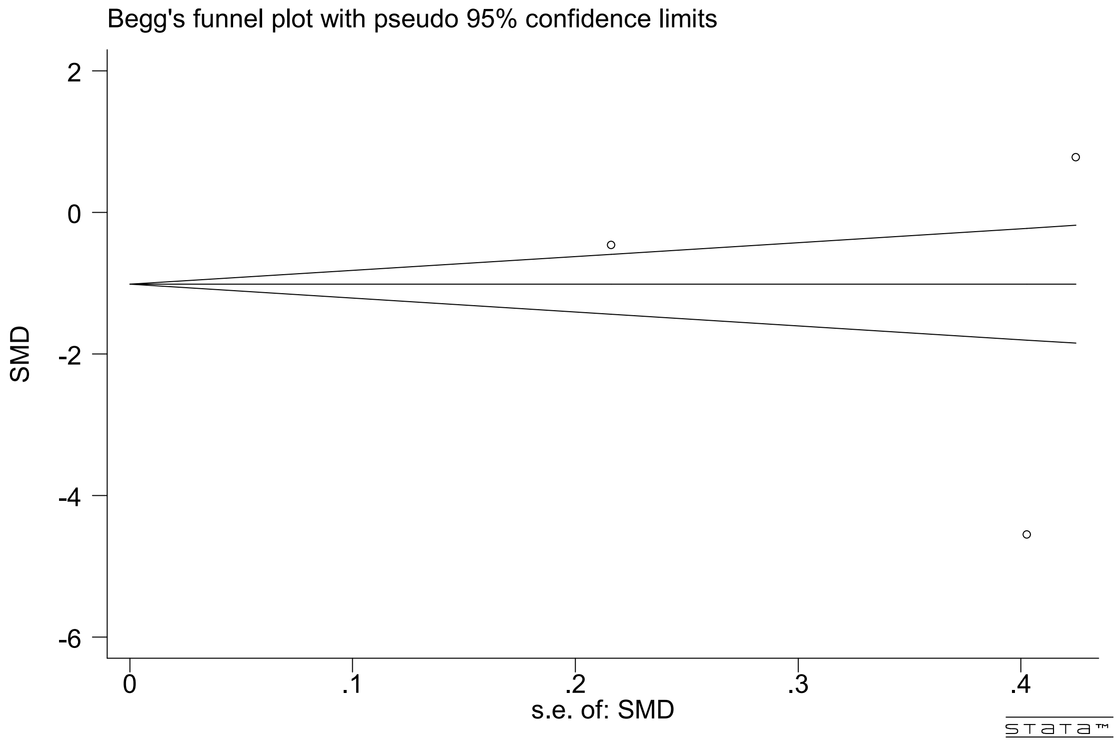

Begg's funnel plot with pseudo 95% confidence limits
2
0
-2
-4
-6
SMD
0
.1
.2
.3
.4
s.e. of: SMD
stata™

(g)
post-treatment vs. pre-treatment
outcome: PI %

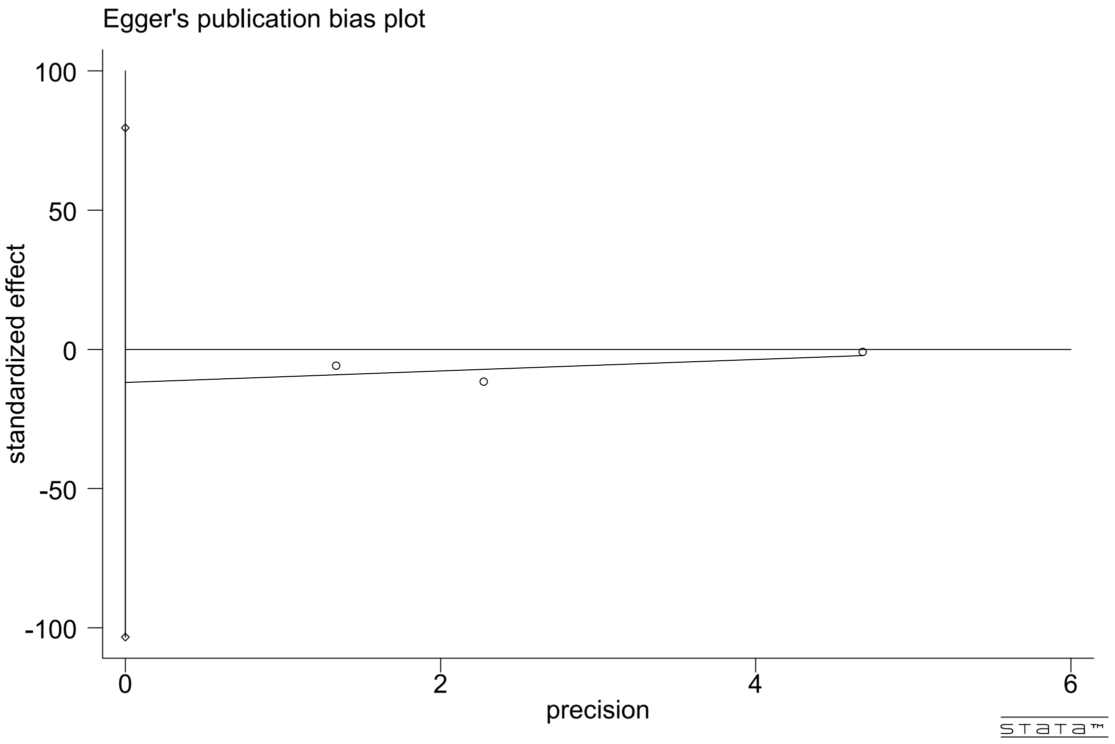

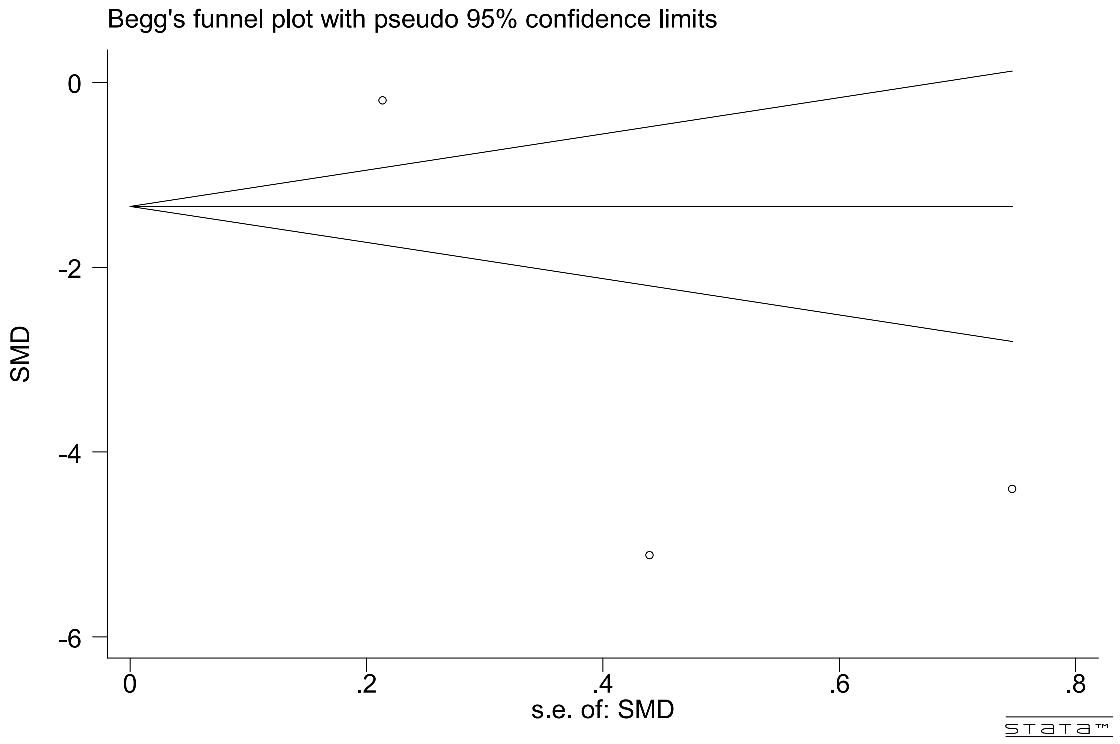
Begg's funnel plot with pseudo 95% confidence limits
0
-2
-4
-6
SMD
0
.2
.4
.6
.8
s.e. of: SMD
stata™

(h)
post-treatment vs. pre-treatment
outcome: PI index

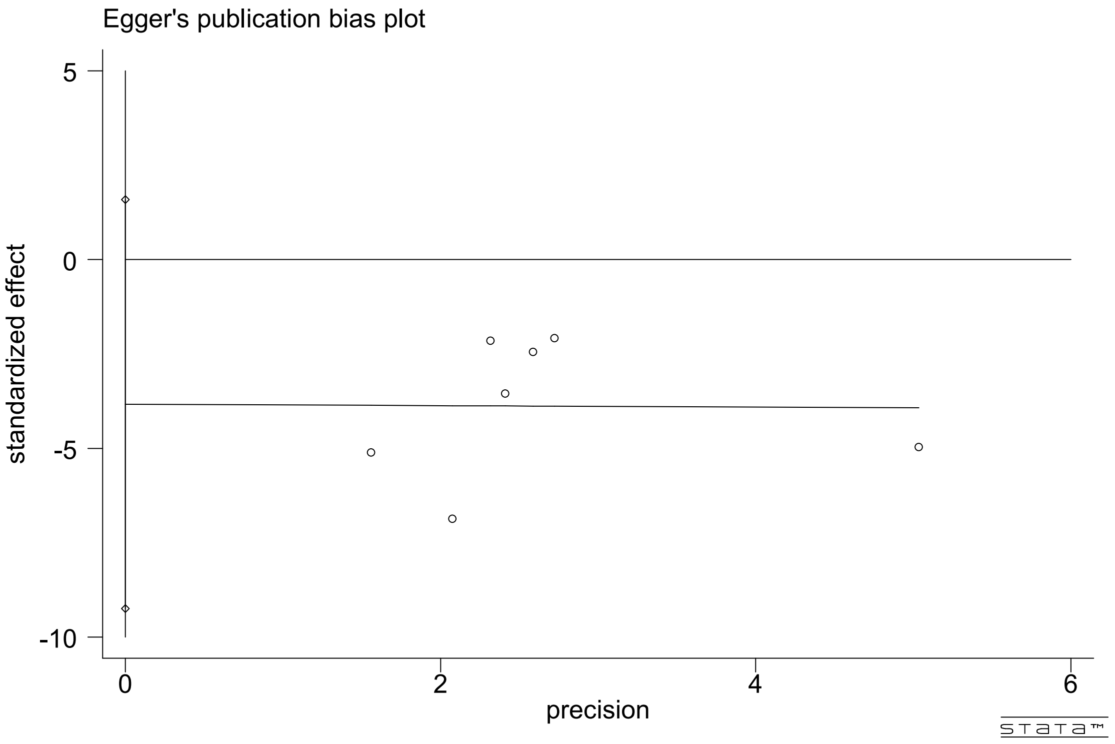

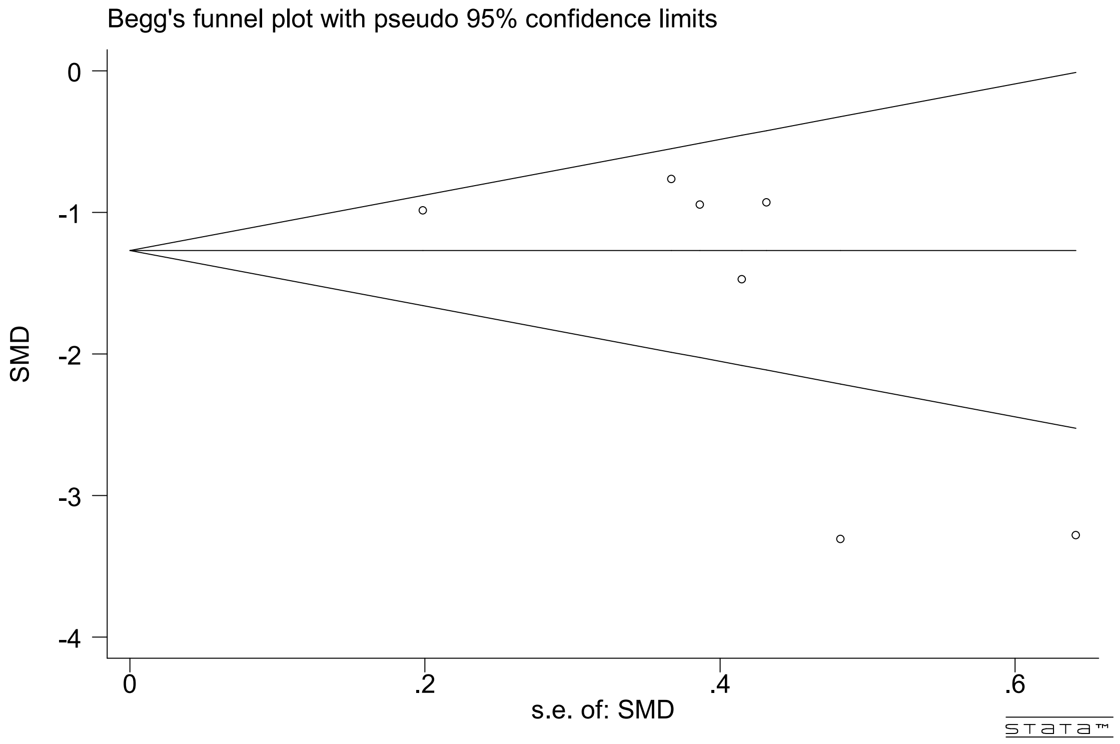
Begg's funnel plot with pseudo 95% confidence limits
0
-1
-2
-3
-4
SMD
0
.2
.4
.6
s.e. of: SMD
stata™
