# Supplementary material for: Non-surgical treatment of cyclosporin A-induced gingival overgrowth: A systematic review and meta-analysis
Source: Medicine (Baltimore). 2025 Jul 18;104(29):e43434. doi: 10.1097/MD.0000000000043434 (PMC12282823; doi:10.1097/MD.0000000000043434)
Supplement: Supplementary file 4 [file medi-104-e43434-s004.pdf]

S3 Table. Meta-analysis of the efficacy of non-surgical periodontal therapy in single group pre-post studies

Outcome	Studies	Cases	SMD (95% CI)	Heterogeneity	
				p	**I^2 (%)**
GO (score)	3	43	-4.17 [-10.07, 1.74]	<0.00001*	98
HI (%)	7	148	-1.49 [-2.92, -0.06]	<0.00001*	96
PD (mm)	7	137	-1.52 [-2.46, -0.58]	<0.00001*	90
PD (%)	3	101	-1.40 [-4.12, 1.32]	<0.00001*	98
PI (%)	3	102	-3.15 [-6.81, 0.51]	<0.00001*	98
PI (index)	7	148	-1.53 [-2.18, -0.88]	<0.00001*	81

Abbreviations: GO, gingival overgrowth; HI, hypertrophy index; PD, probing depth; PI, plaque index.

*Indicates statistical significance, with *p* values less than 0.05.

S4 Table. Summary of effectiveness of non-surgical periodontal treatment by area (Random-Efects Model, SMD)

Type	Outcomes	Classification	Studies	Effect Estimate SMD (95% CI)	I^2 (%)	*p* for heterogeneity
Non-surgical treatment group vs. untreated group						
	PI (index)	Overall	3	-1.06 [-2.04, -0.07]	79	0.009*
		Europe	1	-1.60 [-2.48, -0.71]	/	/
		Asia	2	-0.82 [-2.16, 0.53]	85	0.01*
Before treatment vs. after treatment						
	HI (%)	Overall	7	-1.49 [-2.92, -0.06]	96	<0.00001*
		Europe	3	-1.55 [-4.62, 1.52]	98	<0.00001*
		Asia	3	-1.41 [-1.88, -0.94]	0	0.47
		Africa	1	Not estimable	/	/
	PD (mm)	Overall	7	-1.52 [-2.46, -0.58]	90	<0.00001*
		Europe	2	-1.52 [-2.21, -0.83]	/	/
		Asia	5	-1.56 [-2.68, -0.45]	91	<0.00001*
	PI (index)	Overall	7	-1.53 [-2.18, -0.88]	81	<0.00001*
		Europe	2	-2.06 [-4.36, 0.24]	92	0.0003*
		Asia	5	-1.28 [-1.85, -0.71]	68	0.01*

Abbreviations: PI, plaque index; HI, hypertrophy index; PD, probing depth.

*Indicates statistical significance, with *p* values less than 0.05.

S5 Table. Summary of effectiveness of non-surgical periodontal treatment by gender (Random-Efects Model, SMD)

Type	Outcomes	Classification	Studies	Effect Estimate SMD (95% CI)	I^2 (%)	*p* for heterogeneity
Before treatment vs. after treatment						
	HI (%)	Overall	7	-1.49 [-2.92, -0.06]	96	<0.00001*
		M/F > 1	5	-1.47 [-3.19, 0.24]	97	<0.00001*
		M/F < 1	2	-1.57 [-2.41, -0.74]	96	<0.00001*
	PD (mm)	Overall	7	-1.52 [-2.46, -0.58]	90	<0.00001*
		M/F > 1	6	-1.48 [-2.54, -0.43]	91	<0.00001*
		M/F < 1	1	-1.77 [-2.63, -0.90]	/	/
	PI (index)	Overall	7	-1.53 [-2.18, -0.88]	81	<0.00001*
		M/F > 1	6	-1.65 [-2.41, -0.89]	84	<0.00001*
		M/F < 1	1	-0.92 [-1.68, -0.16]	/	/

Abbreviations: HI, hypertrophy index; PD, probing depth; PI, plaque index; M, male; F, female.

*Indicates statistical significance, with *p* values less than 0.05.

S6 Table. Summary of effectiveness of non-surgical periodontal treatment by antibiotic (Random-Efects Model, SMD)

Type	Outcomes	Classification	Studies	Effect Estimate SMD (95% CI)	I^2 (%)	*p* for heterogeneity
Non-surgical treatment group vs. untreated group						
	PI (index)	overall	3	-1.06 [-2.04, -0.07]	79	0.009*
		yes	1	-0.15 [-0.82, 0.53]	/	/
		no	2	-1.56 [-2.16, -0.96]	0	0.91
Before treatment vs. after treatment						
	GO (score)	overall	3	-4.17 [-10.07, 1.74]	98	<0.00001*
		yes	1	-1.21 [-1.87, -0.54]	/	/
		no	2	-7.23 [-8.97, -5.50]	/	/
	HI (%)	overall	7	-1.49 [-2.92, -0.06]	96	<0.00001*
		yes	5	-2.04 [-3.95, -0.14]	97	<0.00001*
		no	2	-0.38 [-2.98, 2.23]	95	<0.00001*
	PD (mm)	overall	7	-1.52 [-2.46, -0.58]	90	<0.00001*
		yes	4	-1.02 [-1.87, -0.18]	86	<0.00001*
		no	3	-3.57 [-9.28, 2.15]	96	<0.00001*
	PD (%)	overall	3	-1.40 [-4.12, 1.32]	98	<0.00001*
		yes	2	-2.47 [-6.45, 1.50]	99	<0.00001*
		no	1	0.75 [-0.08, 1.58]	/	/
	PI (%)	overall	3	-3.15 [-6.81, 0.51]	98	<0.00001*
		yes	2	-2.62 [-7.40, 2.16]	99	<0.00001*

	no	1	-4.26 [-5.73, -2.79]	/	/
PI (index)	overall	7	-1.53 [-2.18, -0.88]	81	<0.00001*
	yes	3	-0.93 [-1.24, -0.61]	0	0.85
	no	4	-2.14 [-3.30, -0.97]	84	0.0004*

Abbreviations: PI, plaque index; GO, gingival overgrowth; HI, hypertrophy index; PD, probing depth.

*Indicates statistical significance, with *p* values less than 0.05.

S7 Table. Summary of Begg's test and Egger's test

Type	Outcome	Number of studies	Begg's test *p* > |z|	Egger's test *p* > |t|
Non-surgical treatment group vs. untreated group				
	HI (%)	3	1.000	0.774
	PI (index)	3	0.296	0.174
Before treatment vs. after treatment				
	GO (score)	2	1.000	/
	HI (%)	6	0.260	0.390
	PD (mm)	6	0.133	0.003*
	PD (%)	3	1.000	0.762
	PI (%)	3	1.000	0.346
	PI (index)	7	0.016*	0.129

Abbreviations: HI, hypertrophy index; PI, plaque index; GO, gingival overgrowth; PD, probing depth.

*Indicates statistical significance, with *p* values less than 0.05.
